# Broadening the Scope of the ETS-NOCV Scheme: A Versatile Implementation in ORCA

**DOI:** 10.1021/acs.jctc.5c01003

**Published:** 2025-08-07

**Authors:** Ronald Cárdenas Sabando, Christoph Riplinger, Frank Wennmohs, Frank Neese, Giovanni Bistoni

**Affiliations:** † Department of Chemistry, Biology and Biotechnology, University of Perugia, 06123 Perugia, Italy; ‡ FAccTs GmbH, 50677 Koeln, Germany; § Max-Planck-Institut für Kohlenforschung, 45470 Mulheim an der Ruhr, Germany

## Abstract

We report a comprehensive implementation of the Extended Transition State–Natural Orbitals for Chemical Valence (ETS-NOCV) scheme in the orca quantum chemistry package. This implementation broadens the applicability of ETS-NOCV by supporting a wide range of electronic structure methods, including hybrid and double-hybrid density functionals, as well as modern semiempirical and composite approaches such as HF-3c and r^2^SCAN-3c. The implementation is fully parallelized, allowing for the routine analysis of bonding interactions in large molecular systems. We benchmark the method across a set of chemically diverse systems, ranging from noncovalent dimers to dirhodium–carbene complexes, with a particular focus on the performance of semiempirical and double-hybrid functionals, which had not been previously available within this framework. The results highlight the physical interpretability of the method and the computational efficiency of our implementation, providing practical guidelines for selecting appropriate levels of theory in different bonding scenarios.

## Graphical abstract

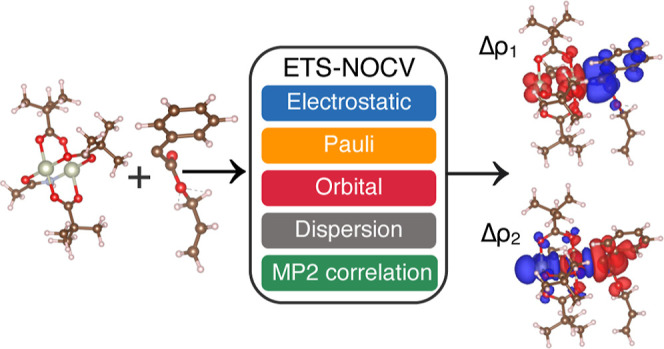

## Introduction

Chemical bonding theory seeks to explain how atoms form molecules, but bonds are not well-defined quantum mechanical observables. As a result, various theoretical frameworks have emerged, each with its own assumptions and limitations, to connect intuitive bonding models with rigorous quantum chemistry.

One major class of bonding schemes focuses on decomposing energy changes during bond formation into chemically meaningful contributions. Originating from the work of Morokuma,^1^ this family includes methods such as the Extended Transition State (ETS) scheme,^2–4^ the Block Localized Wave function Energy Decomposition (BLW-ED),^5^ the Absolutely Localized Molecular Orbital Energy Decomposition Analysis (ALMO-EDA),^6^ the Natural Energy Decomposition Analysis (NEDA),^7^ and the more recent Local Energy Decomposition (LED)^8,9^ at the domain-based local pair natural orbital coupled cluster [DLPNO–CCSD­(T)] level.^10^ Notably, LED is one of the few schemes specifically developed for highly correlated methods that can be applied to systems with hundreds of atoms.

Another prominent family of schemes examines the rearrangement of electron density during bond formation, providing insight into bond order, bond orbitals, and the rearrangement of electron density upon bond formation. Examples of methods in this category include bond order orbitals,^11,12^ natural bond orbitals (NBO),^13–15^ atom in molecules (AIM),^16^ the Electron Localization Function (ELF),^17^ the Laplacian of the electron density (∇_ρ_^2^),^18^ the deformation density (Δρ),^19^ population schemes,^20,21^ and Charge Decomposition Analysis (CDA).^22–24^

An approach that integrates energy decomposition with electron density rearrangement analysis is the Natural Orbitals for Chemical Valence (NOCV) method,^25,26^ when combined with Ziegler’s Extended Transition State (ETS) scheme.^2^ This combined framework, known as ETS-NOCV or Energy Decomposition Analysis with NOCV (EDA-NOCV),^27^ enables a detailed, orbital-based interpretation of specific chemical interactions contributing to bond dissociation energies between molecular fragments.

Later developments have further extended the EDA-NOCV framework. For instance, Sagan and Mitoraj^28^ decomposed the orbital interaction term into kinetic and potential energy contributions, providing a deeper physical interpretation of bonding. Moreover, Parafiniuk and Frenking^29^ proposed an analogous set of orbitals inspired by the ETS-NOCV method to analyze and decompose Pauli repulsion in greater detail.

By design, the ETS-NOCV scheme is formulated within the context of mean-field approaches, such as Hartree–Fock and density functional theory (DFT), and no implementations have been available so far for correlated wave function-based methods.

In this work, we review the theory underlying the ETS-NOCV method and present a general implementation within the ORCA quantum chemistry package,^30^ significantly extending its applicability. In particular, we generalize the scheme to support a wide range of exchange–correlation functionals, including double-hybrid functionals that incorporate a perturbative MP2-like correlation term. This extension allows ETS-NOCV analyses at a higher level of theory than previously possible. Additionally, we enable ETS-NOCV analyses for a range of efficient composite and semiempirical methods, including HF-3c,^31^ PBEh-3c,^32^ B97–3c,^33^ r2SCAN-3c,^34^ and ωB97X-3c.^35^ All features introduced here will be made freely available to the community in the upcoming ORCA 6.1 release.

We conclude by benchmarking the newly enabled ETS-NOCV variants across a diverse set of systems featuring different bonding motifs, highlighting their chemical interpretability and computational efficiency.

## Theory Aspects

This section presents the theoretical foundations of the ETS-NOCV analysis and its implementation in the ORCA code.

While the theoretical aspects of ETS and ETS-NOCV are well established, we begin with a brief and self-contained account here for completeness and ease of reading. This discussion provides the necessary context to understand the specific implementation of ETS-NOCV within the ORCA program. Specifically, the section starts with an overview of the Extended Transition State (ETS) approach, which provides a physically motivated decomposition of the interaction energy between molecular fragments. We then examined the concept of Natural Orbitals for Chemical Valence (NOCVs), which allow for a compact and chemically meaningful representation of the orbital interaction terms and associated electron density rearrangement channels. These two components are subsequently combined to yield the ETS-NOCV scheme.

In the final part of this section, we describe the application of ETS-NOCV scheme to double-hybrid density functionals, and its integration with the 3c family of semiempirical methods.

### Extended Transition State Method

Consider the formation of a chemical bond between two arbitrarily defined fragments, A and B, in their respective equilibrium electronic and geometric configurations. These fragments may represent functional groups, molecular subunits, or entire molecules. Upon interaction, they form a molecular adduct AB. The total binding energy, Δ*E*, quantifies the strength of this interaction and is defined within the supramolecular approach as

1 $$\Delta E=E_{AB}-E_{A}-E_{B}$$

where *E*_AB_, *E*_A_, and *E*_B_ are the total electronic energies of the adduct and of the isolated fragments, respectively.

Within the extended transition state (ETS) scheme,^36^ Δ*E* is further decomposed into chemically meaningful and physically interpretable components. As a first step, Δ*E* is partitioned into two main contributions: the geometric preparation energy (Δ*E*_prep_) and the interaction energy (Δ*E*_int_).

The term Δ*E*_prep_ accounts for the energy required to deform each fragment from its relaxed geometry to the configuration it adopts in the adduct. In contrast, Δ*E*_int_ describes the interaction between the fragments, held frozen in the geometries they exhibit within the complex. This interaction energy is further decomposed as

2 $$\Delta E_{\mathrm{int}}=\Delta E_{elstat}+\Delta E_{Pauli}+\Delta E_{orb}+\Delta E_{disp}$$

where Δ*E*_elstat_ corresponds to the classical electrostatic interaction between the unperturbed charge distributions of the fragments, Δ*E*_Pauli_ reflects the repulsive contribution associated with the overlap between the occupied orbitals of the unperturbed fragments, Δ*E*_orb_ captures the stabilizing effects of orbital relaxation, including charge transfer and polarization, and Δ*E*_disp_ represents the contribution from dispersion interactions.

In summary, the total binding energy is expressed as

3 $$\Delta E=\Delta E_{prep}+\Delta E_{elstat}+\Delta E_{Pauli}+\Delta E_{orb}+\Delta E_{disp}$$

Each term corresponds to a distinct and chemically interpretable step in the bond formation process, as discussed in the following sections.

### Preparation

Energy Δ*E*_prep_. The process begins with the fragments *A* and *B* in their isolated geometric and electronic ground states, characterized by Kohn–Sham wave functions Ψ_*A*_^0^ and Ψ_*B*_^0^, and corresponding electron densities ρ_*A*_^0^(**r**) and ρ_*B*_^0^(**r**). The first step involves distorting the geometries of the fragments from their equilibrium geometries to those they adopt in the adduct *AB*. The associated energy cost is defined as the geometric preparation energy, calculated as

4 $$\Delta E_{prep}=E\left[\rho_{A}\left(r\right)\right]+E\left[\rho_{B}\left(r\right)\right]-E\left[\rho_{A}^{0}\left(r\right)\right]-E\left[\rho_{B}^{0}\left(r\right)\right]$$

here, ρ_*A*_(**r**) and ρ_*B*_(**r**) denote the electron densities of the geometrically distorted fragments, associated with the Kohn–Sham determinants Ψ_*A*_ and Ψ_*B*_. The square brackets indicate that the energy is a functional of the electron density, as is the case in mean-field theories such as DFT.

### Electrostatic Energy Δ*E*_elstat_

In the next step, the geometrically distorted fragments are brought together in the positions they occupy within the adduct, without allowing relaxation of their electron densities. The energy change associated with this step is given by

5 $$E\left[\rho_{A}\left(r\right)+\rho_{B}\left(r\right)\right]-E\left[\rho_{A}\left(r\right)\right]-E\left[\rho_{B}\left(r\right)\right]=\Delta E_{elstat}+\Delta E_{XC}^{0}$$

The term Δ*E*_elstat_ represents the classical electrostatic interaction between the frozen charge distributions of fragments *A* and *B*, including all pairwise Coulomb interactions: between electrons and nuclei of different fragments, between the nuclei of *A* and *B*, and between their electron densities. Explicitly

6 $$\begin{array}{ll} \Delta E_{elstat}= & \sum_{X\in A}\int \frac{\rho_{B}\left(r\right)Z_{X}}{\left|R_{X}-r\right|}dr+\sum_{Y\in B}\int \frac{\rho_{A}\left(r\right)Z_{Y}}{\left|R_{Y}-r\right|}dr\\ & +\sum_{X\in A}\;\sum_{Y\in B}\frac{Z_{X}Z_{Y}}{\left|R_{X}-R_{Y}\right|}+\iint \frac{\rho_{A}\left(r_{1}\right)\rho_{B}\left(r_{2}\right)}{\left|r_{1}-r_{2}\right|}dr_{1}dr_{2} \end{array}$$

here, *Z*_*X*_ and *Z*_*Y*_ denote the nuclear charges of atoms *X* and *Y*, located at positions **R**_*X*_ and **R**_*Y*_ in fragments *A* and *B*, respectively. The first and second terms represent the attraction between nuclei of one fragment and the electron density of the other. The third term accounts for the nuclear repulsion between fragments, and the fourth is the classical Coulomb repulsion between their electron densities.

The second contribution in eq 5, Δ*E*_XC_^0^, accounts for the change in exchange–correlation energy arising from the nonlinear dependence of the Kohn–Sham exchange–correlation functional on the total electron density

7 $$\Delta E_{XC}^{0}=E_{XC}\left[\rho_{A}\left(r\right)+\rho_{B}\left(r\right)\right]-E_{XC}\left[\rho_{A}\left(r\right)\right]-E_{XC}\left[\rho_{B}\left(r\right)\right]$$

At this stage, the total energy *E*[ρ_*A*_(**r**) + ρ_*B*_(**r**)] is evaluated using a wave function given by the product of the individual Kohn–Sham determinants, Ψ_*A*_Ψ_*B*_, with no relaxation of the molecular orbitals upon interaction.

### Pauli Repulsion Energy Δ*E*_Pauli_

The product wave function Ψ_*A*_Ψ_*B*_ does not satisfy the antisymmetry requirement for Fermions. To correct this, it is antisymmetrized and renormalized as follows

8 $$\Psi^{0}=NÂ\left(\Psi_{A}\Psi_{B}\right)$$

where Â is the antisymmetrization operator and *N* is a normalization factor.

For single-determinant systems, this procedure involves orthogonalizing the occupied orbitals of the fragments. This step, implemented using Schmidt orthogonalization,^37^ ensures compliance with the Pauli exclusion principle and results in an increase in the system’s energy

9 $$\Delta {Ẽ}_{Pauli}=E\left[\rho^{0}\left(r\right)\right]-E\left[\rho_{A}\left(r\right)+\rho_{B}\left(r\right)\right]$$

where ρ^0^(**r**) is the electron density associated with Ψ^0^.

It is important to emphasize that $\Delta {Ẽ}_{Pauli}$ differs from Δ*E*_Pauli_ in eq 3. To recover the full Pauli, or “exchange repulsion,” contribution, it is common to add the change in Kohn–Sham exchange–correlation energy Δ*E*_*XC*_^0^

10 $$\Delta E_{Pauli}=\Delta {Ẽ}_{Pauli}+\Delta E_{XC}^{0}$$

As argued in ref 27 these two terms can be combined, as $\Delta {Ẽ}_{Pauli}$ typically dominates over Δ*E*_*XC*_^0^. The term $\Delta {Ẽ}_{Pauli}$ is always positive and destabilizing, reflecting the increase in kinetic energy associated with the formation of the chemical bond.

In certain chemical contexts, the terms Δ*E*_Pauli_ and Δ*E*_elstat_ are grouped together into the so-called Δ*E*_steric_ term.^3,4^ This represents the total energy change resulting from the interaction of two fragments with “frozen” electron densities. The value of Δ*E*_steric_ is generally positive for neutral fragments forming covalent bonds. However, for charged fragments and/or at long interfragment distances, the electrostatic contribution Δ*E*_elstat_ may dominate, potentially changing the sign of Δ*E*_steric_.

### Orbital Energy Δ*E*_orb_

The final step in the energy decomposition allows the electron density of the promolecule, ρ^0^(**r**), to relax into the ground-state density of the fully bonded molecule, ρ­(**r**). The deformation density associated with this relaxation process is defined as

11 $$\Delta \rho \left(r\right)=\rho \left(r\right)-\rho^{0}\left(r\right)$$

This relaxation leads to the orbital interaction energy, Δ*E*_orb_, which captures effects such as donor–acceptor interactions between occupied orbitals of one fragment and virtual orbitals of the other (i.e., charge transfer), as well as intrafragment polarization. Mathematically, this term is defined as

12 $$\Delta E_{orb}=E\left[\rho \left(r\right)\right]-E\left[\rho^{0}\left(r\right)\right]$$

To approximate this energy difference, one can invoke Transition State theory, originally proposed by Slater,^38^ which enables the evaluation of energy differences between electronic states by introducing a fictitious intermediate density, the so-called transition-state density. Although TS theory was initially developed for the calculation of ionization and excitation energies, it was later extended by Ziegler and Rauk^2^ to bond formation and dissociation processes within the Kohn–Sham DFT framework.

In a finite atomic orbital basis set, Δ*E*_orb_ can be approximated by a first-order expansion as

13 $$\Delta E_{orb}\approx \sum_{\mu \nu }\left(F_{\mu \nu }\left[\rho^{TS}\left(r\right)\right]\right)\Delta P_{\mu \nu }=Tr\left\{F^{TS}\Delta P\right\}$$

here, **F** ^TS^ is the Kohn–Sham Fock matrix evaluated at the transition-state density, $\rho^{TS}\left(r\right)=\frac{1}{2}\left[\rho \left(r\right)+\rho^{0}\left(r\right)\right]$ , and Δ ***P*** = **P** – **P** ^0^ is the change in the density matrix associated with the deformation density. *F*_μν_[ρ^TS^(**r**)] and Δ*P*_μν_ denote matrix elements of **F** ^TS^ and Δ**P**, respectively, and μ, ν are atomic orbital indices.

A more accurate expression, also introduced by Ziegler and Rauk,^2^ reduces the truncation error and reads

14 $$\Delta E_{orb}\approx \sum_{\mu \nu }\left(\frac{2}{3}F_{\mu \nu }\left[\rho^{TS}\left(r\right)\right]+\frac{1}{6}F_{\mu \nu }\left[\rho \left(r\right)\right]+\frac{1}{6}F_{\mu \nu }\left[\rho^{0}\left(r\right)\right]\right)\Delta P_{\mu \nu }$$

This second-order expression remains linear in the density matrix change, Δ*P*, and forms the basis for computing orbital interaction energies within the ETS scheme, as implemented in the ORCA program.^36^ As discussed below, this formulation enables the decomposition of Δ*E*_orb_ into chemically meaningful components within the ETS-NOCV framework.

### Dispersion Energy Δ*E*_disp_

Dispersion interactions play a crucial role in determining binding energies and are responsible for a wide range of chemical phenomena across diverse areas of chemical research.^39^

Depending on the chosen exchange—correlation functional, the total binding energy may also include a contribution from the so-called dispersion correction, denoted Δ*E*_disp_. This term arises from an empirical, force-field-like component that is typically added to the DFT energy expression when using standard functionals that lack a proper description of long-range dynamical correlation effects, such as London dispersion interactions. As such, Δ*E*_disp_ provides insight into the change in dispersion energy upon bond formation. Furthermore, the Atomic Decomposition of London Dispersion energy (ADLD), implemented in ORCA, can be used to further decompose Δ*E*_disp_ into contributions from individual atoms, offering chemically intuitive insights into the role of dispersion in molecular recognition, reactivity, and structural preferences.^40,41^

When using doubly hybrid functionals, an extra term corresponding to the second-order Møller–Plesset (MP2) correlation energy is included in the orbital interaction energy. This additional contribution will be discussed in more detail in the section on doubly hybrids.

### Natural Orbitals for Chemical Valence

In the Nalewajski–Mrozek theory of valence and bond orders,^42^ the chemical valence of a closed-shell system is expressed as the expectation value of the so-called valence operator, V̂. Building upon this concept, Mitoraj and Michalak,^43^ and later Mitoraj, Michalak, and Ziegler,^44^ introduced a set of orbitals known as the Natural Orbitals for Chemical Valence (NOCVs) to analyze chemical bonding. The NOCVs are defined as the eigenfunctions that diagonalize the valence operator V̂

15 $$\mathrm{V̂}=\sum_{i=1}^{N}\left(|\psi_{i}^{AB}\left(r\right)\rangle \langle \psi_{i}^{AB}\left(r\right)\left|-|\psi_{i}^{0}\left(r\right)\rangle \langle \psi_{i}^{0}\left(r\right)\right|\right)$$

where *N* is the number of electrons, ψ_*i*_^AB^(**r**) are the occupied spinorbitals of the bonded molecule, and ψ_*i*_^0^(**r**) are the occupied orbitals of the promolecule. The promolecular orbitals are constructed by orthogonalizing the fragment orbitals, obtained from separate self-consistent field (SCF) calculations in which each fragment is kept frozen in the geometry it adopts in the final adduct.

It is important to note that this formulation is inherently defined within a mean-field framework, where the total density is expressed as a simple sum over *N* orbital contributions. As such, it cannot be directly applied to correlated wave function methods without further theoretical developments.

The total valence *V* associated with bond formation is defined as the expectation value of V̂ over the molecular adduct wave function. For closed-shell systems, and assuming a common basis for both the molecular adduct and the promolecule, this leads to the following expression

16 V=⟨Ψ|V̂|Ψ⟩=Tr(PΔP)

where Ψ is the wave function of the adduct, **P** is its one-electron density matrix, and **Δ*P*** = **P** – **P** ^**0**^ is the difference with respect to the promolecule density matrix **P** ^**0**^.

The NOCVs are defined as the eigenfunctions ψ_*i*_(**r**) of the valence operator V̂

17 $$\mathrm{V̂}\psi_{i}\left(r\right)=v_{i}\psi_{i}\left(r\right)$$

where *i* = 1, 2, ..., *n*, and *n* is the number of basis functions. These orbitals provide a one-electron decomposition of the deformation density

18 $$\Delta \rho \left(r\right)=\sum_{i=1}^{n}v_{i}\psi_{i}^{2}\left(r\right)$$

with each term representing a distinct channel of electron density redistribution upon bond formation.

When both the molecular adduct and the promolecule can be described by single Slater determinants, the spectrum of V̂ consists of eigenfunctions appearing in pairs, with eigenvalues of equal magnitude and opposite sign

19 $$\mathrm{V̂}\psi_{k}\left(r\right)=v_{k}\psi_{k}\left(r\right)$$

20 $$\mathrm{V̂}\psi_{-k}\left(r\right)=-v_{k}\psi_{-k}\left(r\right)$$

where *k* = 1, 2, ..., *n*/2. In practice, only a few orbital pairs contribute significantly to the deformation density, corresponding to the largest (in magnitude) eigenvalues. The total deformation density can then be expressed in terms of these paired contributions as

21 $$\Delta \rho \left(r\right)=\sum_{k=1}^{n/2}v_{k}\left[\psi_{k}^{2}\left(r\right)-\psi_{-k}^{2}\left(r\right)\right]=\sum_{k=1}^{n/2}\Delta \rho_{k}\left(r\right)$$

where each term Δρ_*k*_(**r**) represents the charge flow from ψ_–*k*_ to ψ_*k*_, driven by the eigenvalue *v*_*k*_.

This decomposition corresponds to the diagonalization of the density difference matrix, Δ ***P***, which yields the eigenvectors **c**_*k*_ that define the NOCVs in a given one-electron orbital basis.

## The Combined ETS-NOCV Scheme

In their 2009 study, Mitoraj, Michalak, and Ziegler^27^ introduced the use of the orthonormal basis defined by the NOCVs to express the orbital interaction energy (eq 14), as

22 $$\Delta E_{orb}=\sum_{k}^{n/2}\left({\mathrm{F̃}}_{kk}-{\mathrm{F̃}}_{-k-k}\right)\nu_{k}=\sum_{k=1}^{n/2}\Delta E_{orb,k}$$

where

23 $${\mathrm{F̃}}_{kk}=\frac{2}{3}F_{kk}\left[\rho^{TS}\left(r\right)\right]+\frac{1}{6}F_{kk}\left[\rho \left(r\right)\right]+\frac{1}{6}F_{kk}\left[\rho^{0}\left(r\right)\right]$$

This expression provides a chemically intuitive decomposition of the orbital interaction energy, Δ*E*_orb_, into pairwise contributions from individual NOCV channels, denoted as Δ*E*_orb,*k*_

In summary, the ETS-NOCV decomposition establishes a direct connection between visualizable charge-transfer channels and their energetic contributions to bonding. Specifically, each NOCV pair {ψ_*k*_, ψ_–*k*_} represents a distinct mode of electron flow between (or within) fragments, characterized by the associated deformation density Δρ_*k*_(**r**). The corresponding energy contribution, Δ*E*_orb,*k*_, quantifies the stabilizing (or destabilizing) effect of this specific interaction on the overall bonding energy.

## Functional-Dependent Evaluation of the ETS-NOCV Energy Terms

The ETS-NOCV scheme can be applied at various levels of theory, and it is therefore essential to clarify how each energy term is evaluated depending on the type of exchange–correlation functional or the approximations employed.

In Kohn–Sham DFT, the total electronic energy of a molecular system is given by

24 $$E_{tot}=T_{s}+V_{ext}\left[\rho \left(r\right)\right]+J\left[\rho \left(r\right)\right]+E_{XC}\left[\rho \left(r\right)\right]$$

where *T*_*s*_ is the kinetic energy of noninteracting electrons, *V*_ext_[ρ­(**r**)] denotes the electron–nucleus interaction energy, *J*[ρ­(**r**)] is the classical Coulomb repulsion between electrons, and *E*_XC_[ρ­(**r**)] is the exchange–correlation energy, which also implicitly accounts for the missing portion of the kinetic and electron–electron correlation energy.

In the following, we describe how the ETS-NOCV scheme is adapted for specific classes of exchange–correlation functionals, including (i) hybrid DFT functionals incorporating a fraction of Hartree–Fock (HF) exchange, (ii) double-hybrid functionals that incorporate MP2-like correlation, and (iii) dispersion-corrected semiempirical methods such as those in the “3c” family. Particular attention is given to how the exchange–correlation term Δ*E*_XC_^0^ is evaluated in each case, to ensure internal consistency with the underlying electronic structure model.

### Hybrid Functionals

For hybrid functionals, the exchange–correlation energy incorporates a fraction of HF exchange, and it is typically written as^45^

25 $$E_{XC}^{\left(hybrid\right)}=aE_{X}^{HF}+\left(1-a\right)E_{X}^{DFT}+E_{C}^{DFT}$$

where *a* is the mixing coefficient, *E*_X_^HF^ is the Hartree–Fock exchange, *E*_X_^DFT^ is the exchange contribution from a pure DFT functional, and *E*_C_^DFT^ is the corresponding DFT correlation energy.

When evaluating the ETS-NOCV energy components, the exchange–correlation term Δ*E*_XC_^0^ is computed using *E*_XC_ for pure functionals and *E*_XC_^hybrid^ for hybrid functionals.

### Double Hybrid Functionals

This class of functionals operates essentially like a hybrid-DFT method, augmented by an MP2 correlation energy contribution, *E*_C_^MP2^, computed using DFT orbitals and added to the final energy. This MP2 term aims to improve the description of electron correlation effects,^46^ and is therefore included in the exchange–correlation energy

26 $$E_{XC}^{\left(double‐hybrid\right)}=a_{X}E_{X}^{HF}+\left(1-a_{X}\right)E_{X}^{DFT}+\left(1-a_{C}\right)E_{C}^{DFT}+a_{C}E_{C}^{MP2}$$

Double-hybrid functionals are generally defined by the empirical parameters *a*_*C*_ and *a*_*X*_, as well as by the choice of exchange and correlation functionals. In the case of B2PLYP, used for the illustrative calculations in this article, the Becke 88 exchange functional is used for *E*_X_^DFT^, the Lee–Yang–Parr (LYP) functional for *E*_C_^DFT^, and the empirical parameters are set to *a*_*X*_ = 0.53 and *a*_*C*_ = 0.27.^47^

It is important to note that, for double hybrids, the electron density differs from that of a standard DFT calculation, as it results from a correlated method. Specifically, the density matrix is no longer an idempotent projector, and its eigenvalues lie between 0 and 1 (or between 0 and 2 in the restricted KS case for closed shell molecules). As a result, the usual pairing conditions for the eigenvalues of **Δ*P*** are not fulfilled, making it impossible to perform a standard NOCV decomposition without approximations.

To address this issue, we exploited the fact that the deformation density obtained with a double hybrid functional, Δρ^(double‑hybrid)^(**r**), can be expressed as the sum of a “pure” DFT contribution and a relaxed (or unrelaxed) MP2 correction

27 $$\Delta \rho^{\left(double‐hybrid\right)}\left(r\right)=\Delta \rho^{DFT}\left(r\right)+\Delta \Delta \rho_{C}^{MP2}\left(r\right)$$

here, Δρ^DFT^(**r**) denotes the deformation density obtained from the SCF procedure using the double-hybrid functional, while ΔΔρ_C_^MP2^(**r**) is simply defined as

28 $$\Delta \Delta \rho_{C}^{MP2}\left(r\right)=\Delta \rho^{\left(double‐hybrid\right)}\left(r\right)-\Delta \rho^{DFT}\left(r\right)$$

The physical meaning of ΔΔρ_C_^MP2^(**r**) is to describe how the inclusion of MP2 correlation influences the rearrangement of electron density associated with bond formation. As will be shown numerically below, this correction is typically small in standard chemical applications, but it can nonetheless be analyzed in detail within the present theoretical framework.

In our implementation, the NOCVs are obtained by considering only the electron density derived from the pure DFT part of the calculation. This ensures that the eigenvalue pairing property of the NOCVs is preserved. However, ΔΔρ_C_^MP2^(**r**) can still be analyzed and visualized, and its contribution to the total energy can be readily estimated. Specifically, the energy associated with the MP2 correlation term is printed in ORCA as an additional component of the EDA decomposition, labeled MP2 correlation. This corresponds to the difference in the a_C_E_C_^MP2^ term between the full system and the isolated fragments.

### Semiempirical Methods

The 3c family. Semiempirical “3c” methods combine a minimal quantum-mechanical core with three empirical corrections—dispersion, basis-set superposition error, and short-range basis deficiencies—to deliver accurate geometries, frequencies, and noncovalent interaction energies at low computational cost. In ORCA, the available variants are HF-3c,^31^ PBEh-3c,^32^ B97–3c,^33^ r2SCAN-3c,^34^ and wB97x-3c.^35^ All of these “3c” methods are supported in the ORCA implementation of the ETS-NOCV scheme, yielding reasonable binding-energy decompositions at a fraction of the cost of fully converged DFT or higher-level methods. Each “3c” recipe differs only in (i) the exchange–correlation functional and basis set used for the QM core and (ii) the parametrization (and sometimes the type) of the three empirical corrections.

Below, we show the expression for the HF-3c energy as a representative example

29 $$E_{tot}^{HF‐3c}=E_{tot}^{HF/MINIX}+E_{disp}+E_{BSSE}^{gCP}+E_{SRB}$$

here, *E*_tot_^HF/MINIX^ is the energy from a Hartree–Fock calculation using the minimal MINIX basis set. It provides a simple QM backbone but lacks electron-correlation effects and suffers from basis-set deficiencies. *E*_disp_ is Grimme’s atom-pairwise D3 dispersion correction with Becke–Johnson damping,^48–51^ which recovers long-range van der Waals interactions (London dispersion) absent in HF or standard XC functionals. *E*_BSSE_^gCP^ is the geometrical counterpoise correction of Kruse et al.,^52^ removing artificial overbinding caused by basis-set superposition error (BSSE). *E*_SRB_ (short-range basis correction) compensates for the tendency of small/minimal basis sets to overestimate bond lengths—especially involving electronegative atoms—by adding a pairwise, exponentially decaying penalty term.^31^ Other “3c” variants follow the same four-term structure but use different functionals and basis sets, and reparametrize (or omit) the empirical corrections accordingly.

When performing ETS-NOCV in ORCA with any 3c method, the semiempirical corrections are treated as separate binding-energy terms. Since they are computed for both fragments and complex, they can be cleanly included as extra EDA contributions. For HF-3c, one computes

30 $$\Delta E_{\mathrm{int}}^{HF‐3c}=\Delta E_{\mathrm{int}}^{HF/MINIX}+\Delta E_{disp}+\Delta E_{BSSE}^{gCP}+\Delta E_{SRB}$$

The Δ*E*_int_^HF–3c^ term can be further decomposed within the ETS-NOCV scheme into Δ*E*_elstat_, Δ*E*_Pauli_, and Δ*E*_orb_, as described in the previous sections. The latter can, in turn, be further resolved into NOCV contributions. The Δ*E*_disp_ represents the dispersion contribution to the interaction, consistent with the discussion above. The remaining two terms, Δ*E*_BSSE_^gCP^ and Δ*E*_SRB_, both account for basis set deficiencies. These are therefore grouped together into a single correction term. This approach retains the standard decomposition into electrostatic, Pauli repulsion, orbital (charge-transfer), and dispersion interactions, while simultaneously providing insight into how basis set limitations affect the total binding energy.

## Extension to Open-Shell Systems

For open-shell systems, such as radicals or transition metal complexes with unpaired electrons, the direct application of the NOCV decomposition as described for closed-shell cases encounters a fundamental limitation: the one-electron density matrices **P** and **P** ^**0**^ are no longer idempotent. This loss of idempotency arises from the spin-unrestricted nature of the underlying SCF solutions, where the α and β spin densities are treated independently. As a result, the difference matrix Δ ***P*** = **P** – **P** ^**0**^ does not generally yield paired eigenvalues of equal magnitude and opposite sign, and hence, its eigenfunctions cannot be used to define a consistent set of NOCVs.

However, a meaningful ETS-NOCV decomposition can still be performed by treating the spin-resolved density matrices independently, as described in the original works of Mitoraj, Michalak, and Ziegler,^27^ and Mitoraj and Michalak.^26^ For each spin component, σ = α, β, the deformation density matrix is defined as

31 $$\Delta P^{\sigma }=P^{\sigma }-P^{0,\sigma }$$

which is guaranteed to be the difference of two idempotent matrices, each derived from a single-determinant wave function. Thus, within each spin channel, the valence operator remains well-defined

32 $${\mathrm{V̂}}^{\sigma }=\sum_{i=1}^{N^{\sigma }}\left(|\psi_{i}^{\sigma }\rangle \langle \psi_{i}^{\sigma }\left|-|\psi_{i}^{0,\sigma }\rangle \langle \psi_{i}^{0,\sigma }\right|\right)$$

where ψ_*i*_^σ^(**r**) are the occupied spin orbitals for the spin-σ channel, *N* ^σ^ is the number of electrons with spin $\sigma \psi_{i}^{0,\sigma }\left(r\right)$ denoting the orthogonalized fragment spin orbitals. The corresponding deformation densities can be express as

33 $$\Delta \rho^{\sigma }\left(r\right)=\sum_{i=1}^{n_{\sigma }}v_{i}^{\sigma }\psi_{i}^{\sigma ,2}\left(r\right)$$

where the ψ_*i*_^σ^ are the spin-resolved NOCVs and *v*_*i*_^σ^ are the associated eigenvalues of the spin-specific valence operator ${\mathrm{V̂}}^{\sigma }$ .

As in the closed-shell case, the spin-resolved deformation density can be expressed in terms of paired eigenfunctions

34 $$\Delta \rho^{\sigma }\left(r\right)=\sum_{k=1}^{n_{\sigma }/2}v_{k}^{\sigma }\left[\psi_{k}^{\sigma ,2}\left(r\right)-\psi_{-k}^{\sigma ,2}\left(r\right)\right]=\sum_{k=1}^{n_{\sigma }/2}\Delta \rho_{k}^{\sigma }\left(r\right)$$

The total deformation density is obtained by summing the contributions from both spin channels

35 $$\Delta \rho \left(r\right)=\Delta \rho^{\alpha }\left(r\right)+\Delta \rho^{\beta }\left(r\right)$$

Similarly, the orbital interaction energy can be decomposed into spin-resolved ETS-NOCV contributions

36 $$\Delta E_{orb}=\sum_{\sigma =\alpha ,\beta }\;\sum_{k=1}^{n_{\sigma }/2}\Delta E_{orb,k}^{\sigma }$$

where each term Δ*E*_orb,*k*_^σ^ quantifies the energetic contribution of the *k*-th spin-specific charge transfer channel.

This formulation preserves the interpretability and chemical insight provided by the ETS-NOCV scheme to systems with unpaired electrons. Each spin channel contributes independently to the overall deformation density and orbital interaction energy, allowing for a clear, spin-resolved picture of the bonding process.

## Computational Details

All calculations presented in this study were performed with a development version of the orca program package, based on version 6.0.^53^ The density functional approximations employed include BLYP,^54,55^ B3LYP,^45^ and B2PLYP,^47^ corresponding to a pure, hybrid, and double-hybrid functional, respectively. Unless otherwise stated, the def2-QZVP basis set^56^ was used throughout. To account for dispersion interactions, the D3 correction with Becke–Johnson (BJ)^48,57^ damping was applied in all cases.

For the semiempirical calculations, the following 3c methods were employed: HF-3c^31^ and r^2^SCAN-3c.^34^

All molecular structures used in these calculations are provided in the Supporting Information, along with the results of the basis set convergence tests. The latter were performed using the def2 basis set family (def2-SVP, def2-TZVP, def2-TZVP­(−*f*), def2-QZVP)^56^ for each system and functional.

## Illustrative Case Studies

To showcase the performance and practical utility of ETS-NOCV, we present a series of case studies that allow a systematic comparison of different electronic structure methods for different bonding motifs. These examples highlight not only the physical insights accessible through ETS-NOCV but also the relative strengths and limitations of various density functionals and semiempirical approaches (e.g., HF-3c) in capturing the different bonding contributions with acceptable accuracy and computational cost.

### Small Molecular Dimers with Distinct Interaction Types

In this section, we present the results of the ETS-NOCV decomposition for a selected set of small molecular systems characterized by different types of intermolecular interactions.^58^ The systems considered are the argon dimer Ar_2_, the argon–lithium cation complex Ar–Li^+^, the beryllium dimer Be_2_, the hydrogen fluoride dimer (HF)_2_, and the water dimer (H_2_O)_2_, as shown in Figure 1.

**Figure 1:**
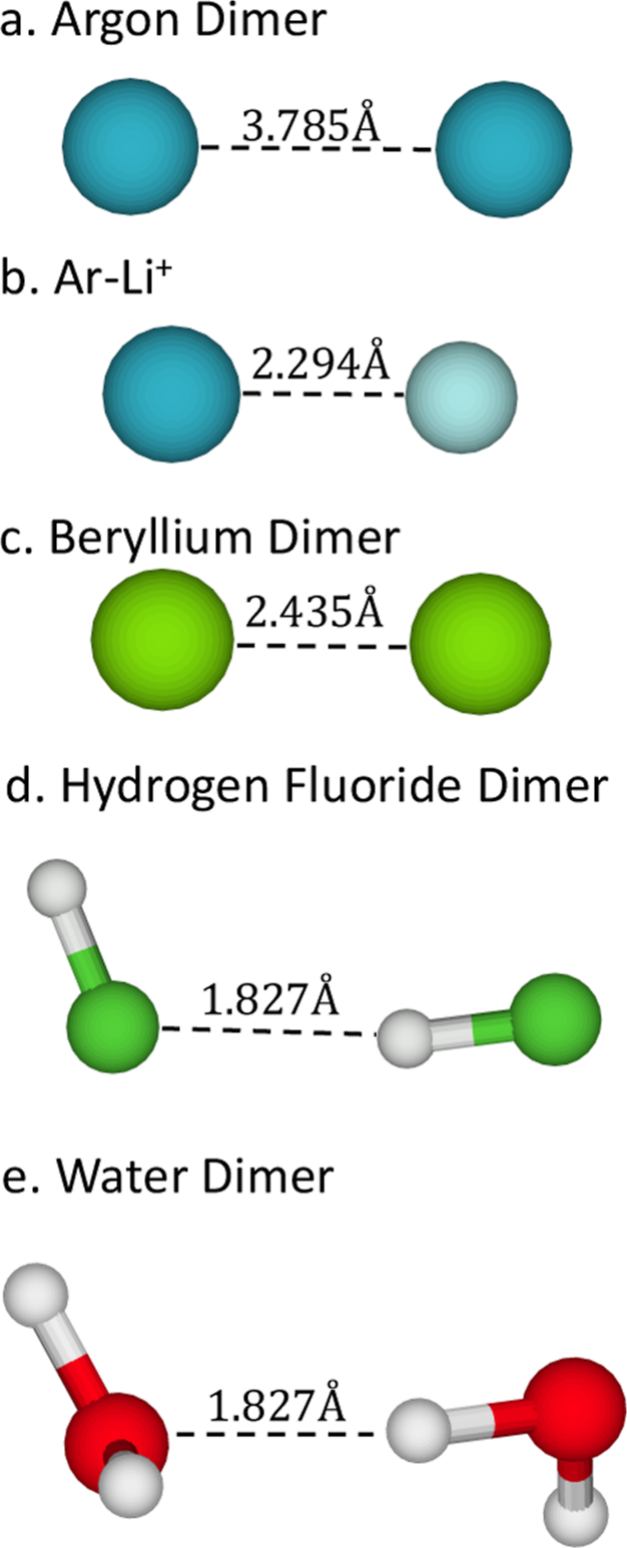
Studied molecules with intermolecular distances.

While a combination of effects is responsible for the overall interaction in each system, a dominant physical driving force can be identified in each case. In the case of the argon dimer, the interaction is mainly governed by London dispersion forces,^59^ which arise from correlated instantaneous dipole fluctuations. For the Ar–Li^+^ complex, the interaction is dominated by charge-induced electrostatic effects, where the electric field associated with the lithium cation polarizes the electron density of the neutral argon atom.^60^ In contrast, the bonding in the beryllium dimer is primarily characterized by weak covalent (electron-sharing) interactions, with a delicate balance between bonding and antibonding orbital contributions.^61^ Finally, both the hydrogen fluoride and water dimers are prototypical hydrogen-bonded systems, in which the dominant contribution to the interaction energy originates from classical electrostatics, with additional stabilization arising from polarization and charge-transfer effects. These systems thus provide a chemically diverse test set for validating the ORCA implementation of the ETS-NOCV scheme, particularly its application across a wide range of electronic structure methods—including double-hybrid functionals and semiempirical approaches—that were not previously available in this context. This allows for a consistent analysis of different bonding regimes, ranging from dispersion-dominated to electrostatic and covalent interactions.

Table 1 reports the results of the ETS decomposition for the Ar_2_ dimer at various levels of theory.

**Table 1:** ETS Analysis for Ar–Ar at different Levels of Theory Using Various Functionals, Including a Pure Functional (BLYP), a Hybrid Functional (B3LYP), and a Double-Hybrid Functional (B2PLYP)^a^

	BLYP-D3(BJ)	B3LYP-D3(BJ)	B2PLYP-D3(BJ)	HF–3C	r2SCAN-3C
Δ*E*_int_	–0.15	–0.21	–0.18	–0.07	–0.21
Δ*E*_orb_	–0.05	–0.04	–0.03	–0.01	–0.04
Δ*E*_Pauli_	0.59	0.55	0.68	0.34	0.03
Δ*E*_elstat_	–0.18	–0.29	–0.47	–0.11	–0.12
Δ*E*_disp_	–0.52	–0.43	–0.22	–0.42	–0.09
Δ*E*_C_^MP2^			–0.14		
Δ*E*_BSSE_^gCP^+Δ*E*_SRB_				0.13	0.02

a All DFT calculations include the D3 dispersion correction with Becke–Johnson damping. The def2-QZVP basis set was employed for all calculations. Additionally, results from 3c composite and semiempirical methods are reported.

As expected for noble gas interactions, the binding in Ar_2_ is dominated by London dispersion forces. In most cases, dispersion (Δ*E*_disp_) emerges as the most stabilizing contribution, ranging from −0.52 kcal/mol with BLYP-D3 to −0.09 kcal/mol with r^2^SCAN-3C.

At first glance, electrostatic interactions (Δ*E*_elstat_) may appear significant, particularly for B2PLYP-D3, where they reach values as large as −0.47 kcal/mol. However, this contribution is fully compensated by the Pauli repulsion term (Δ*E*_Pauli_), resulting in a net repulsive steric interaction (i.e., Δ*E*_Pauli_ + Δ*E*_elstat_) that remains positive and relatively stable across all methods.

As expected for interactions between closed-shell noble gases, orbital relaxation effects (Δ*E*_orb_) play only a minor role and contribute negligibly to the total interaction energy. The NOCV decomposition of this term (Figure 2) reveals two weak, counterbalancing contributions corresponding to the population of both bonding and antibonding orbitals between the two argon atoms, in line with classical molecular orbital theory. While all electronic structure methods yield qualitatively similar deformation densities, the magnitude of charge displacement varies significantly across methods. In particular, HF-3c shows the smallest degree of charge rearrangement, as evidenced by its lower *v*_*k*_ eigenvalues.

**Figure 2:**
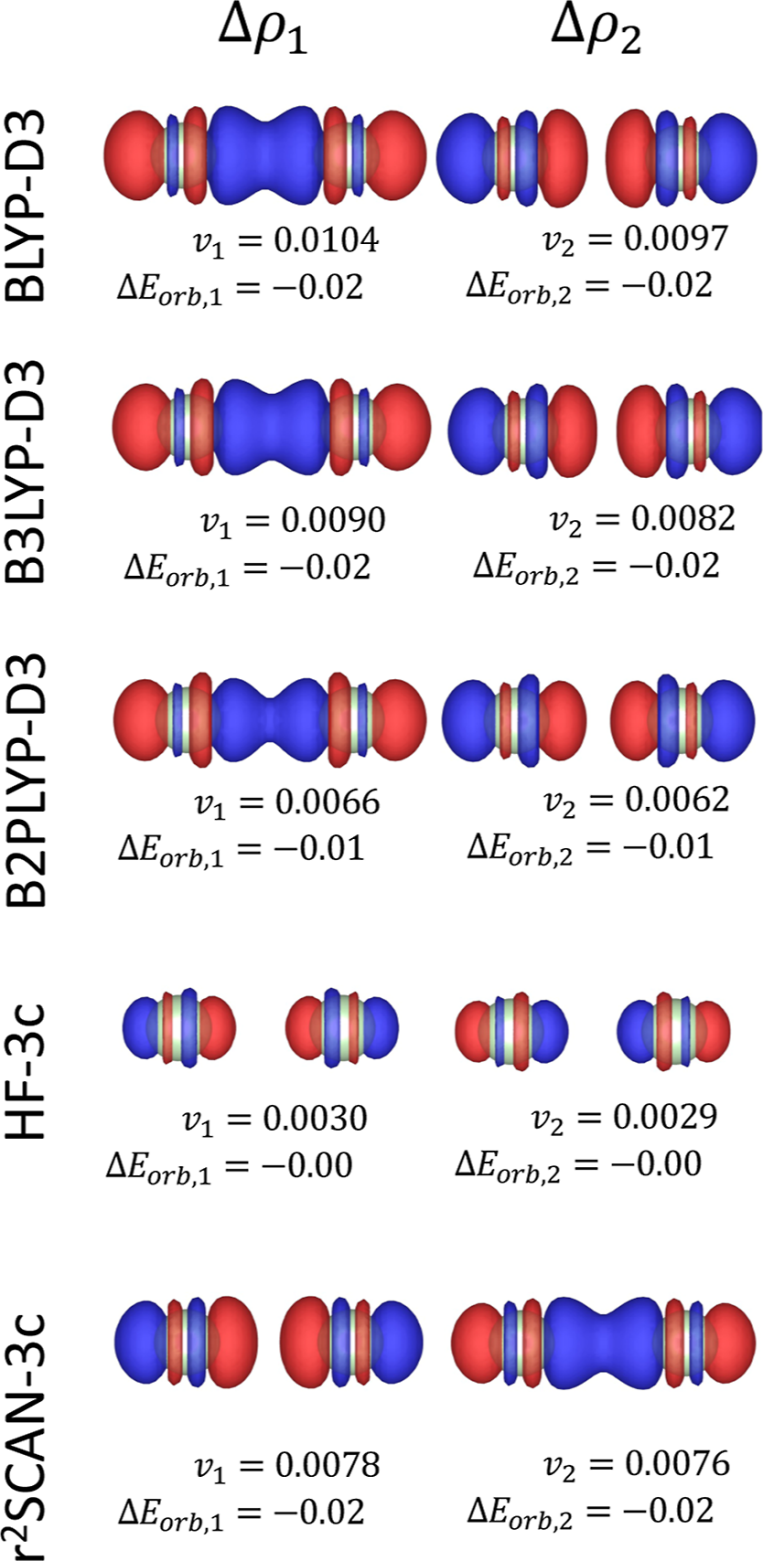
First two NOCV deformation densities Δρ_1_ (left panels) and Δρ_2_ (right panels) for the Ar–Ar system for different electronic structure methods. The corresponding NOCV eigenvalues (*v*_1_, *v*_2_) and orbital interaction energy contributions (Δ*E*_orb,1_, Δ*E*_orb,2_) are shown below each panel. The energy contributions are express in kcal/mol. All deformation densities are visualized at an isosurface level of 2 × 10^–5^ e/Bohr^3^. Red and blue surfaces indicate regions of charge depletion and accumulation, respectively.

These results confirm the well-established picture of noble gas interactions: Ar_2_ can be described as a weakly interacting pair of nearly spherical atoms, with minimal polarization and strong short-range repulsion. The only attractive force is the long-range dispersion interaction, which is entirely responsible for the formation and stability of the dimer in the gas phase. Importantly, these results remain generally valid across all tested methods.

In Table 2 we can see the ETS-NOCV results for the interaction of Ar–Li^+^.

**Table 2:** ETS Analysis for Ar–Li^+^ at different Levels of Theory Using Various Functionals, Including a Pure Functional (BLYP), a Hybrid Functional (B3LYP), and a Double-Hybrid Functional (B2PLYP)^a^

	BLYP-D3(BJ)	B3LYP-D3(BJ)	B2PLYP-D3(BJ)	HF–3C	r2SCAN-3C
Δ*E*_int_	–8.33	–8.13	–7.60	–2.26	–4.89
Δ*E*_orb_	–11.66	–11.33	–10.87	–4.43	–9.04
Δ*E*_Pauli_	4.18	4.41	5.02	4.42	4.44
Δ*E*_elstat_	0.06	–0.44	–1.17	–0.54	–0.28
Δ*E*_disp_	–0.92	–0.77	–0.41	–1.94	–0.09
Δ*E*_C_^MP2^			–0.17		
Δ*E*_BSSE_^gCP^+Δ*E*_SRB_				0.23	0.00

a All DFT calculations include the D3 dispersion correction with Becke–Johnson damping. The def2-QZVP basis set was employed for all calculations. Additionally, results from 3c composite and semiempirical methods are reported.

Finally, Unlike the Ar–Ar dimer, the interaction in Ar–Li^+^ is dominated by induction (polarization) effects rather than by pure dispersion. Given that argon possesses a spherical electron density with no permanent multipole moments, the electrostatic interaction with the positively charged lithium ion is expected to be weak. This expectation is confirmed by the results obtained with all functionals. Although the electrostatic term (Δ*E*_elstat_) is relatively large in absolute value for double-hybrid functionals, this increase is entirely compensated by a concomitant increase in Pauli repulsion (Δ*E*_Pauli_), such that the net steric contribution remains small and consistent across methods.

For this system, the orbital interaction term (Δ*E*_orb_) emerges as the dominant attractive component, ranging from −11.66 kcal/mol (BLYP-D3) to −4.43 kcal/mol (HF–3C). For all the tested electronic structure methods, the NOCV decomposition of Δ*E*_orb_ (Figure 3) reveals two primary contributions, corresponding to the polarization of the σ- and π-type electron densities of the argon atom toward the lithium cation.

**Figure 3:**
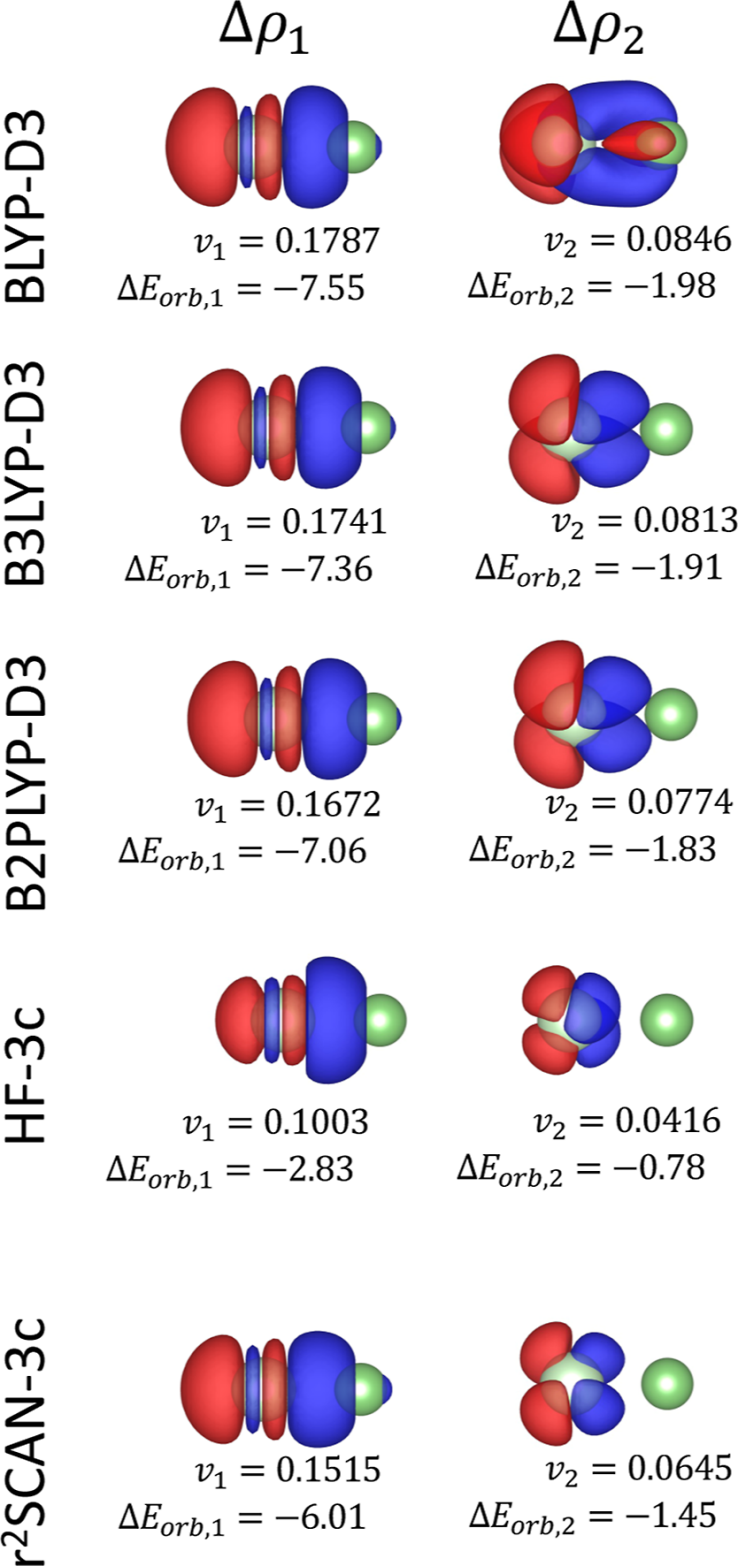
First two NOCV deformation densities Δρ_1_ (left panels) and Δρ_2_ (right panels) for the Ar–Li^+^ system for different electronic structure methods. The corresponding NOCV eigenvalues (*v*_1_, *v*_2_) and orbital interaction energy contributions (Δ*E*_orb,1_, Δ*E*_orb,2_) are shown below each panel. The energy contributions are express in kcal/mol. All deformation densities are visualized at an isosurface level of 2 × 10^–4^ e/Bohr^3^. Red and blue surfaces indicate regions of charge depletion and accumulation, respectively.

The bonding in Be_2_ has been widely studied,^61^ since the beryllium atom features a closed-shell electronic configuration (1s^2^2s^2^), which—according to simple molecular orbital theory—should not support bonding between two neutral atoms. Nonetheless, Be_2_ has been shown to form a weakly bound dimer in the gas phase,^62^ exhibiting a covalent interaction driven by subtle electron sharing. The ETS-NOCV decomposition for Be_2_ is reported in Table 3.

**Table 3:** ETS Analysis for Be–Be at different Levels of Theory Using Various Functionals, Including a Pure Functional (BLYP), a Hybrid Functional (B3LYP), and a Double-Hybrid Functional (B2PLYP)^a^

	BLYP-D3(BJ)	B3LYP-D3(BJ)	B2PLYP-D3(BJ)	HF–3C	r2SCAN-3C
Δ*E*_int_	–8.72	–6.23	–3.40	12.18	–8.04
Δ*E*_orb_	–32.04	–29.40	–25.65	–10.56	–30.77
Δ*E*_Pauli_	43.84	55.69	78.12	54.37	41.51
Δ*E*_elstat_	–17.91	–30.35	–51.53	–26.21	–18.47
Δ*E*_disp_	–2.61	–2.17	–1.11	–5.44	–0.26
Δ*E*_*C*_^*MP2*^			–3.24		
Δ*E*_BSSE_^gCP^+Δ*E*_SRB_				0.02	0.00

a All DFT calculations include the D3 dispersion correction with Becke–Johnson damping. The def2-QZVP basis set was employed for all calculations. Additionally, results from 3c composite and semiempirical methods are reported.

Among the methods considered, only HF-3c predicts a positive total interaction energy (+12.18 kcal/mol), indicating a failure to describe bonding. This result reflects a known limitation of Hartree–Fock-based methods, which lack dynamic correlation and thus cannot account for the subtle electron sharing responsible for bonding in Be_2_. This limitation is also evident from the comparatively large dynamic correlation correction (Δ*E*_C_^MP2^) observed for the double-hybrid functional B2PLYP-D3­(BJ). Indeed, among the model systems examined in this work, Be_2_ is the only one for which HF-3c exhibits a qualitative failure. As expected, the key stabilizing term in all correlated methods is the orbital interaction energy (Δ*E*_orb_), which ranges from −32.04 kcal/mol (BLYP-D3­(BJ)) to −25.65 kcal/mol (B2PLYP-D3­(BJ)), while HF-3c severely underestimates this contribution (−10.56 kcal/mol).

This discrepancy is also evident in the NOCV analysis, where the deformation density associated with the first NOCV pair in HF-3c is notably weaker compared to that shown at the B3LYP level (Figure 4).

**Figure 4:**
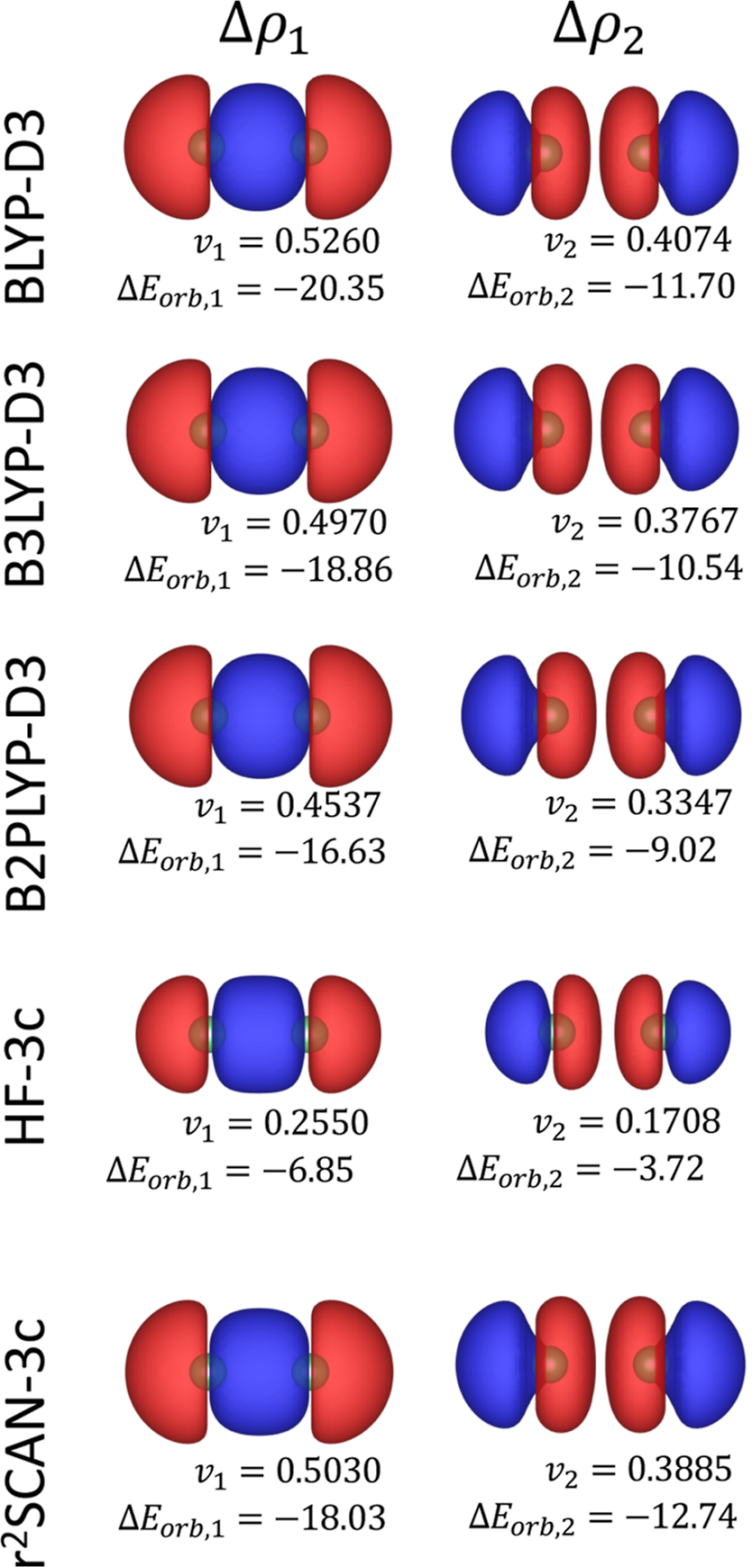
First two NOCV deformation densities Δρ_1_ (left panels) and Δρ_2_ (right panels) for the Be–Be system for different electronic structure methods. The corresponding NOCV eigenvalues (*v*_1_, *v*_2_) and orbital interaction energy contributions (Δ*E*_orb,1_, Δ*E*_orb,2_) are shown below each panel. The energy contributions are express in kcal/mol. All deformation densities are visualized at an isosurface level of 5 × 10^–4^ e/Bohr^3^. Red and blue surfaces indicate regions of charge depletion and accumulation, respectively.

Specifically, the first NOCV pair in HF-3c contributes only Δ*E*_orb,1_ = −6.85 kcal/mol, whereas the corresponding values for BLYP and B3LYP are Δ*E*_orb,1_ = −20.35 and −18.86 kcal/mol, respectively. These results highlight the inability of HF-3c to capture the covalent character of the Be–Be bond, which arises primarily from mixing of the 2s orbitals. Dispersion contributions are modest across all methods (maximum of −2.61 kcal/mol for BLYP-D3), consistent with previous findings that van der Waals forces play a minor role in Be_2_ binding.^58^

The r2SCAN-3c method also performs reasonably well, predicting an interaction energy of −8.04 kcal/mol and a first NOCV contribution of Δ*E*_orb,1_ = −18.03 kcal/mol. Overall, the ETS-NOCV analysis confirms that bonding in Be_2_ is dominated by orbital interactions, which are significantly enhanced by electron correlation. Methods that neglect dynamic correlation, such as HF-3c, fail to capture this delicate balance, whereas double-hybrid functionals and *meta*-GGAs succeed in reproducing the essential physics of the bond.

In Table 4 the ETS analysis for (HF)_2_ is reported. This is a prototypical hydrogen-bonded system in which electrostatics and orbital interactions drive stability.

**Table 4:** ETS-NOCV Analysis for (HF)_2_ at different Levels of Theory Using Various Functionals, Including a Pure Functional (BLYP), a Hybrid Functional (B3LYP), and a Double-Hybrid Functional (B2PLYP)^a^

	BLYP-D3(BJ)	B3LYP-D3(BJ)	B2PLYP-D3(BJ)	HF–3C	r2SCAN-3C
Δ*E*_int_	–4.53	–4.87	–4.75	–0.93	–4.28
Δ*E*_orb_	–3.47	–3.13	–2.66	–1.57	–3.15
Δ*E*_Pauli_	6.16	6.89	8.56	6.68	4.84
Δ*E*_elstat_	–6.76	–8.26	–10.35	–8.74	–6.88
Δ*E*_disp_	–0.45	–0.37	–0.16	–0.96	–0.07
Δ*E*_C_^MP2^			–0.15		
Δ*E*_BSSE_^gCP^+Δ*E*_SRB_				3.66	0.98

a All DFT calculations include the D3 dispersion correction with Becke–Johnson damping. The def2-QZVP basis set was employed for all calculations. Additionally, results from 3c composite and semiempirical methods are reported.

The electrostatic term (Δ*E*_elstat_) is the dominant stabilizing force, varying from −6.76 kcal/mol (BLYP-D3) to −10.35 kcal/mol (B2PLYP-D3), with HF-3c predicting a value of −8.74 kcal/mol, in line with those obtained from more sophisticated approaches. Orbital interactions (Δ*E*_orb_) provide additional stabilization, ranging from −3.47 kcal/mol (BLYP-D3) to −1.57 kcal/mol (HF-3c), with the primary interaction corresponding to charge transfer from the fluorine lone pair to the adjacent hydrogen atom (Figure 5). Pauli repulsion is significant, ranging from +4.84 to +8.56 kcal/mol, and is particularly pronounced in B2PLYP-D3, conssitent with the trends discussed above. HF-3c also underestimates the total binding energy (Δ*E*_int_ = −0.93 kcal/mol), reflecting its limited treatment of the subtle charge-transfer and polarization effects that characterize this system. Dispersion interactions are negligible, ranging from −0.45 kcal/mol (BLYP-D3) to −0.07 kcal/mol (r2SCAN-3c), confirming that the stabilization of (HF)_2_ is not dispersion-driven.

**Figure 5:**
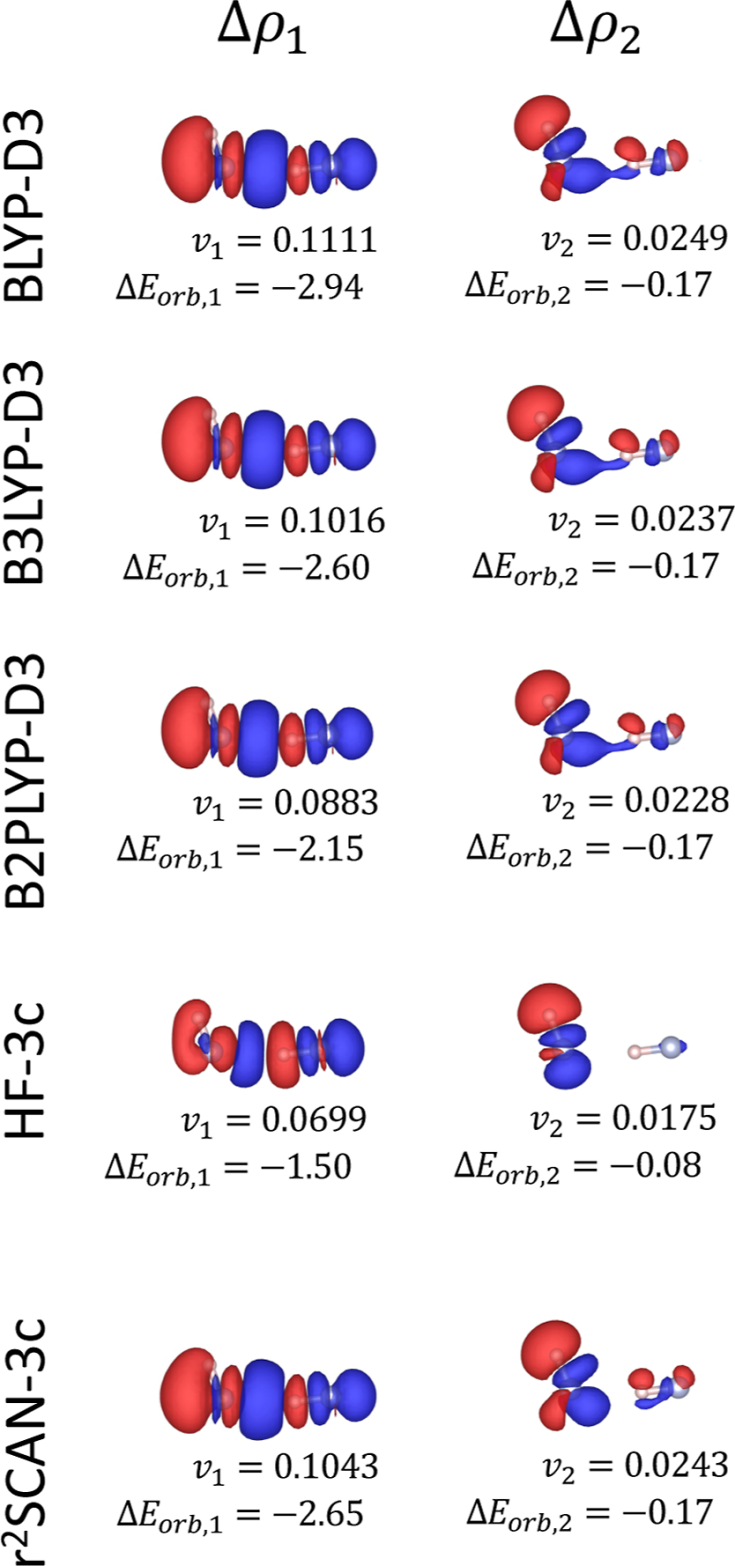
First two NOCV deformation densities Δρ_1_ (left panels) and Δρ_2_ (right panels) for the HF–HF system for different electronic structure methods. The corresponding NOCV eigenvalues (*v*_1_, *v*_2_) and orbital interaction energy contributions (Δ*E*_orb,1_, Δ*E*_orb,2_) are shown below each panel. The energy contributions are express in kcal/mol. All deformation densities are visualized at an isosurface level of 2 × 10^–4^ e/Bohr^3^. Red and blue surfaces indicate regions of charge depletion and accumulation, respectively.

Similarly, the water dimer represents a prototypical hydrogen-bonded system, sharing many characteristics with the (HF)_2_ but exhibiting even stronger electrostatic interactions. As shown in Table 5, the electrostatic component (Δ*E*_elstat_) remains the dominant stabilizing force, ranging from −8.40 kcal/mol (BLYP-D3) to −13.61 kcal/mol (B2PLYP-D3). HF-3c yields an electrostatic energy of −9.90 kcal/mol, consistent with values from correlated methods and appropriate for a moderately strong hydrogen bond. Orbital interactions (Δ*E*_orb_) also contribute significantly, ranging from −3.55 kcal/mol (BLYP-D3) to −1.66 kcal/mol (HF-3c), and are associated with lone-pair donation from the oxygen atom into the σ* orbital of the adjacent O–H bond (Figures 6 and 7).

**Table 5:** ETS-NOCV Analysis for the Water Dimer at different Levels of Theory Using Various Functionals, Including a Pure Functional (BLYP), a Hybrid Functional (B3LYP), and a Double-Hybrid Functional (B2PLYP)^a^

	BLYP-D3(BJ)	B3LYP-D3(BJ)	B2PLYP-D3(BJ)	HF–3C	r2SCAN-3C
Δ*E*_int_	–4.85	–5.17	–5.05	–3.86	–4.72
Δ*E*_orb_	–3.55	–3.21	–2.77	–1.66	–3.22
Δ*E*_Pauli_	7.87	9.19	11.99	7.55	6.37
Δ*E*_elstat_	–8.40	–10.52	–13.61	–9.90	–8.39
Δ*E*_disp_	–0.77	–0.63	–0.28	–1.32	–0.14
Δ*E*_C_^MP2^			–0.37		
Δ*E*_BSSE_^gCP^+Δ*E*_SRB_				1.48	0.65

a All DFT calculations include the D3 dispersion correction with Becke–Johnson damping. The def2-QZVP basis set was employed for all calculations. Additionally, results from 3c composite and semiempirical methods are reported.

**Figure 6:**
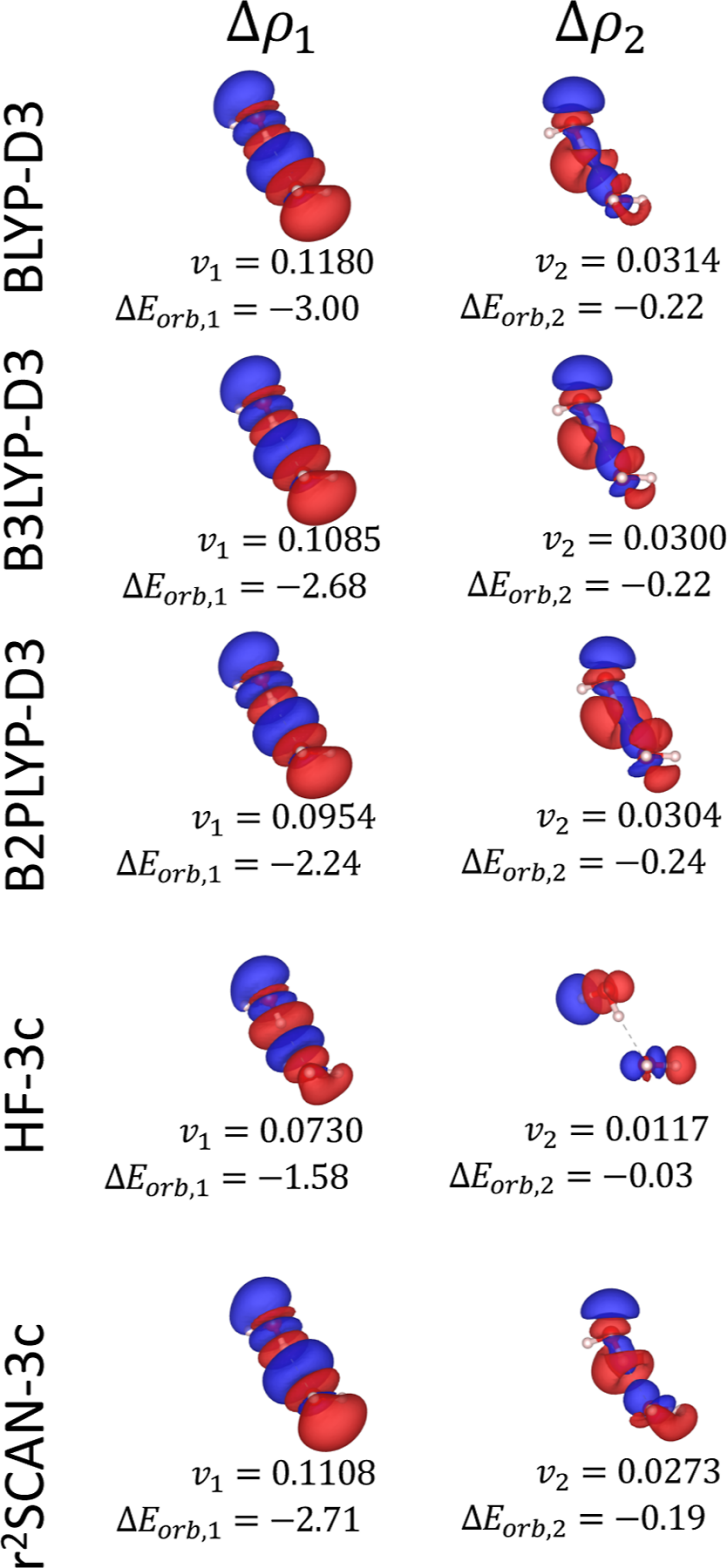
First two NOCV deformation densities Δρ_1_ (left panels) and Δρ_2_ (right panels) for the H_2_O–H_2_O system for different electronic structure methods. The corresponding NOCV eigenvalues (*v*_1_, *v*_2_) and orbital interaction energy contributions (Δ*E*_orb,1_, Δ*E*_orb,2_) are shown below each panel. The energy contributions are express in kcal/mol. All deformation densities are visualized at an isosurface level of 1 × 10^–4^ e/Bohr^3^. Red and blue surfaces indicate regions of charge depletion and accumulation, respectively.

**Figure 7:**
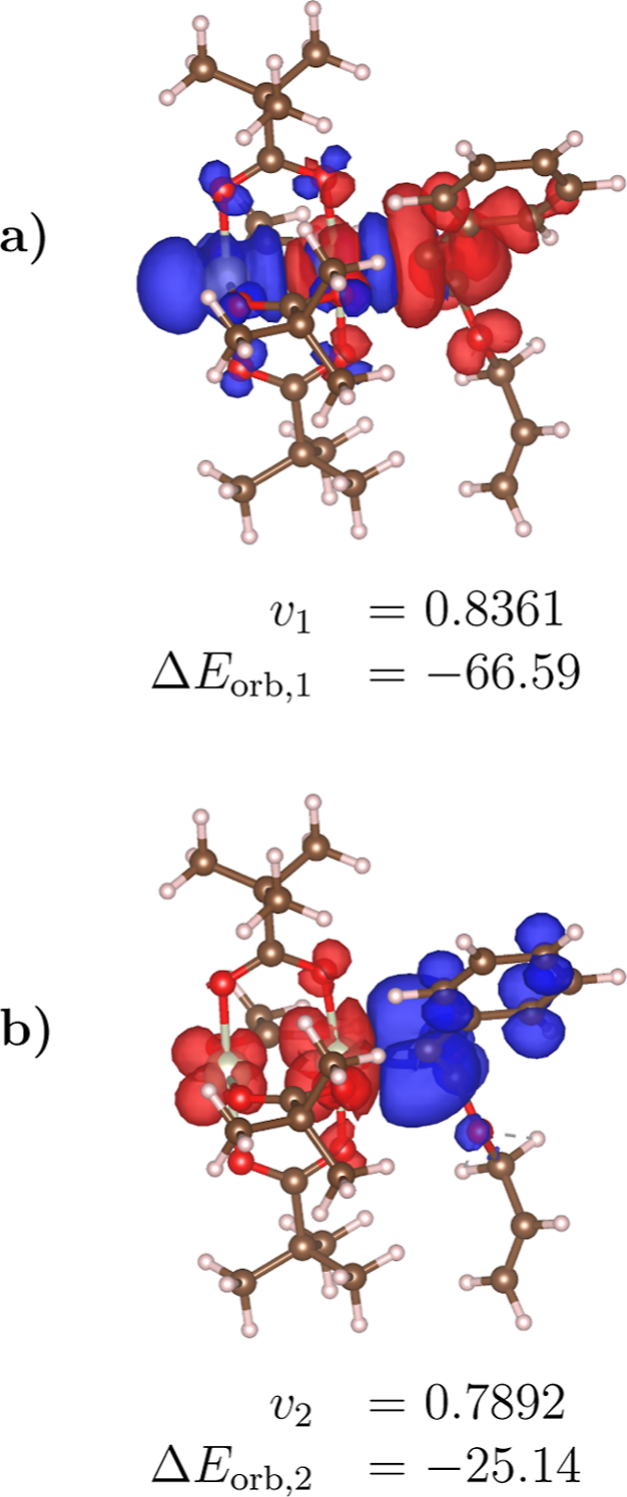
First two NOCV deformation densities Δρ_1_ (panel a) and Δρ_2_ (panel b) for the dirhodium­(II)–carbene system using the B3LYP-D3 functional. The corresponding NOCV eigenvalues (*v*_1_, *v*_2_) and orbital interaction energy contributions (Δ*E*_orb,1_, Δ*E*_orb,2_) are shown below each panel. The energy contributions are expressed in kcal/mol. All deformation densities are visualized at an isosurface level of 5 × 10^–4^ e/Bohr^3^. Red and blue surfaces indicate regions of charge depletion and accumulation, respectively. See Figure S2 for results with other functionals.

Pauli repulsion is comparable to that observed in (HF)_2_, ranging from +6.37 to +11.99 kcal/mol, with B2PLYP-D3 yielding the highest value. Dispersion plays a minor role, contributing less than 1 kcal/mol across all methods, with the exception of HF-3c, where it is somewhat overestimated (−1.32 kcal/mol). As with (HF)_2_, HF-3c underestimates the total interaction energy (Δ*E*_int_ = −3.86 kcal/mol), reflecting the known limitations of Hartree–Fock-based methods in capturing correlation-driven effects in hydrogen bonding. Overall, the ETS-NOCV analysis confirms that electrostatics and orbital charge transfer are the principal contributors to the stability of the water dimer.

### Dirhodium-Carbene Results

The bonding interactions in dirhodium­(II)–carbene complexes play a crucial role in determining the reactivity and selectivity of these widely used catalysts.^63^ These interactions are commonly described as donor–acceptor in nature, involving strong σ-donation from the carbene lone pair into Rh d-orbitals, complemented by π-backdonation from filled Rh d-orbitals into the empty p orbital of the carbene. The ETS decomposition for a prototypical dirhodium­(II)–carbene complex is shown in Table 6.

**Table 6:** ETS-NOCV Analysis for the Bond between Rhodium­(II) and Carbene in a Dirhodium­(II)-Carbene Complex^a^

	BLYP-D3(BJ)	B3LYP-D3(BJ)	B2PLYP-D3(BJ)	HF–3C	r2SCAN-3C
Δ*E*_int_	–67.71	–61.16	–62.45	–36.40	–68.05
Δ*E*_orb_	–121.48	–110.13	–97.81	–84.17	–117.58
Δ*E*_Pauli_	191.94	223.31	285.12	193.54	165.89
Δ*E*_elstat_	–116.07	–155.63	–219.47	–126.93	–111.68
Δ*E*_disp_	–22.09	–18.72	–9.80	–23.81	–5.94
Δ*E*_C_^MP2^			–20.50		
Δ*E*_BSSE_^gCP^+Δ*E*_SRB_				4.98	1.26

a DFT Results are Reported Using Various Functionals, Including a Pure Functional (BLYP), a Hybrid Functional (B3LYP), and a Double-Hybrid Functional (B2PLYP). All DFT calculations include the D3 dispersion correction with Becke–Johnson damping. The def2-QZVP basis set was employed for all calculations. Additionally, results from 3c composite and semiempirical methods are reported.

Across various electronic structure methods, the total interaction energy (Δ*E*_int_) ranges from −68.05 kcal/mol (r^2^SCAN-3c) to −36.40 kcal/mol (HF-3c). Electrostatic interactions (Δ*E*_elstat_) contribute significantly to the overall stabilization, whereas Pauli repulsion (Δ*E*_Pauli_) opposes bond formation, resulting in a net steric contribution that is repulsive. Overall, the analysis confirms that the metal–carbene bond is primarily stabilized by strong orbital interactions (Δ*E*_orb_).

The decomposition of Δ*E*_orb_ within the ETS-NOCV scheme reveals distinct bonding contributions that are consistent with the textbook description of bonding in these systems. The primary orbital interaction (Δ*E*_1_^orb^), associated with σ-donation from the carbene to the metal center, is the most stabilizing component, ranging from −66.59 kcal/mol (B3LYP-D3) to −60.89 kcal/mol (HF-3c) (see Figure S2 in the Supporting Information). Secondary orbital contributions (Δ*E*_2_^orb^) indicate a significant role for π-backdonation, particularly in functionals that better capture electron correlation, such as BLYP-D3­(BJ).

Importantly, the ETS-NOCV implementation in orca described in this work has been fully parallelized, enabling calculations on large systems and/or with large basis sets within a reasonable computational time. Figure S1 presents a parallelization benchmark for the ETS-NOCV analysis of the metal–carbene bond in the dirhodium complex at the B3LYP-D3­(BJ)/def2-QZVP level of theory, as a function of the number of processors.

As a final remark, it is worth discussing the relative efficiency of the electronic structure methods evaluated in this work, with the aim of providing practical guidelines on their applicability. The full computational timings for the ETS-NOCV decomposition are reported in Figure 8. These results clearly demonstrate the substantial gain in computational efficiency when transitioning from conventional exchange–correlation functionals to HF-3c. Moreover, it is evident that double-hybrid functionals are significantly more computationally demanding than the other methods examined. This increase in cost is well-known and is generally accompanied by a marked improvement in accuracy, except in well-documented pathological cases where the underlying MP2 treatment is inadequate.

**Figure 8:**
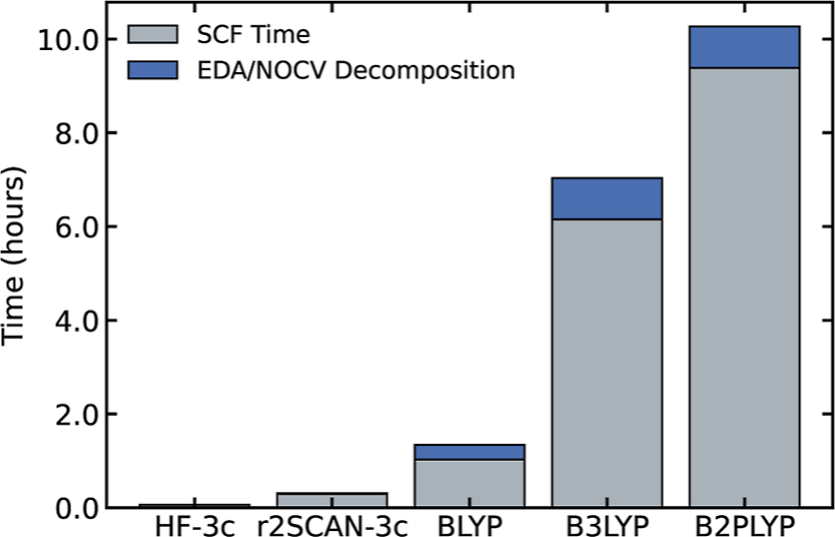
Total run time (in hours) for the complete computation of the dirhodium–carbene system using different electronic structure methods. Each bar is divided into the total SCF run time (bottom segment) and the time specifically required for the ETS-NOCV decomposition (top segment). Calculations with BLYP, B3LYP, and B2PLYP were performed using the def2-QZVP basis set. All methods include the D3 dispersion correction with Becke-Johnson damping. Calculations were carried out using 16 cores of an AMD EPYC 75F3 processor.

Nonetheless, the MP2-level treatment provides valuable insight into how correlation influences the electron density distribution, as illustrated in Figure S3 (Supporting Information). In all systems examined, the correlation density redistributes charge around the bonding region, typically leading to a depletion of electron density in the middle of the bond.

## Implementation Details and Efficiency

The implementation of the ETS-NOCV method in orca relies on evaluating the electron density matrices of both the adduct and the promolecule. As discussed in the theory section, the promolecule represents a hypothetical, noninteracting assembly of the molecular fragments in the geometry they adopt within the adduct, but without allowing for any electronic relaxation.

In orca, the promolecule density matrix is constructed from the density matrices of the isolated fragments. This is achieved by combining the fragment orbitals and subsequently orthonormalizing them via the Schmidt procedure.

An ETS-NOCV calculation therefore involves three separate self-consistent field (SCF) calculations: one for the full adduct and one for each molecular fragment. Additionally, the computation of orbital interaction energies requires evaluation of the Fock operator at three different densities: that of the adduct, the promolecule, and a transition-state-like density. This step is computationally demanding, as building each Fock matrix is equivalent to a full SCF iteration.

Moreover, an extra Fock matrix is constructed to evaluate the $\Delta {Ẽ}_{Pauli}$ term, which involves computing the energy of a reference system whose electron density is the sum of the unrelaxed fragment densities.

Once all relevant density matrices and Fock operators are available, the various ETS terms are computed as energy differences between the adduct, promolecule, reference system, transition state, and fragments. Figure 8 shows the total computational time for each method, with the portion dedicated to the ETS-NOCV decomposition highlighted separately. The ETS-NOCV timing includes only the decomposition analysis itself and does not account for the time required to compute the electronic energies.

The current implementation of ETS-NOCV in orca automates the full sequence of energy evaluations. The user is only required to specify the fragments and methods (level of theory, basis set, etc.); all necessary intermediate steps and calculations are handled internally by the program.

## Conclusions

In this work, we presented a general and efficient implementation of the ETS-NOCV scheme within the ORCA program. This development broadens the applicability of the method in three main directions. First, the implementation is compatible with nearly all density functionals available in ORCA, including hybrid and double-hybrid functionals. Second, the method has been extended to include the 3c family of composite and semiempirical approaches, such as HF-3c and r^2^SCAN-3c, enabling bonding analyses in large molecular systems at low computational cost. Third, the implementation is fully parallelized, allowing ETS-NOCV analyses to be performed efficiently on complex systems using standard computational resources.

We assessed the robustness and consistency of the method across a range of chemically diverse systems—including noble gas dimers, hydrogen-bonded complexes, and a representative dirhodium–carbene complex. The results show that key features of the NOCV decomposition are preserved across different levels of theory, with qualitatively similar bonding patterns observed in all cases. While double-hybrid functionals are known to offer the highest accuracy, especially in systems where dynamic correlation effects are significant, semiempirical approaches such as HF-3c can still provide valuable qualitative insights, particularly for large-scale applications. Nevertheless, caution is warranted when employing such low-cost methods for systems with complex electronic structure.

Overall, the new ETS-NOCV implementation in ORCA offers a versatile and chemically insightful tool for studying bonding interactions across a wide range of systems and theoretical methods.

## Supplementary Material



## Notes

Additional material including: Parallelization benchmark figures; NOCV’s deformation density plots for the dirhodium­(II)–carbene system; basis set convergence tables for all methods; Cartesian coordinates (XYZ format) of polyatomic molecular structures and MP2 correlation electron density contribution for all the studied systems (PDF)

## ∥

Current affiliation: Max Planck Institute for the Physics of Complex Systems, 01187, Dresden, Germany
