# Supplementary material for: Broadening the Scope of the ETS-NOCV Scheme: A Versatile Implementation in ORCA
Source: J Chem Theory Comput. 2025 Aug 7;21(16):7920–34. doi: 10.1021/acs.jctc.5c01003 (PMC12392439; doi:10.1021/acs.jctc.5c01003)
Supplement: Supplementary file 1 [file ct5c01003_si_001.pdf]

Electronic Supporting Information (ESI)

Broadening the Scope of the ETS-NOCV scheme: A Versatile Implementation in ORCA

Ronald Cárdenas Sabando,[†,§] Christoph Riplinger,[‡] Frank Wennmohs ,[¶] Frank Neese ,[¶] and Giovanni Bistoni[*,†]

†*Department of Chemistry, Biology and Biotechnology, University of Perugia, 06123 Perugia, Italy*

‡*FAccTs GmbH, 50677 Koeln, Germany.*

¶*Max-Planck-Institut fu r Kohlenforschung, 45470 Mulheim an der Ruhr, Germany*

§*Current affiliation: Max Planck Institute for the Physics of Complex Systems, 01187, Dresden, Germany*

E-mail: giovanni.bistoni@unipg.it

Parallelization Benchmark

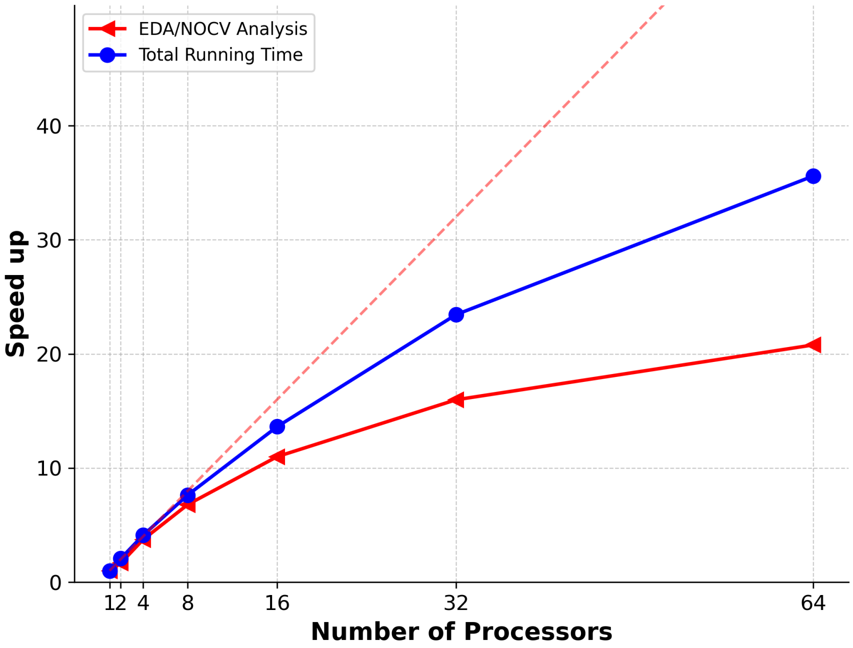

Figure S1: Total run time in minutes for different processor counts for the EDA-NOCV analysis of the dirhodium(II)-carbene at the B3LYP-D3 (BJ)def2-QZVP. Calculations were run on a AMD EPYC 75F3 processor.

Basis Set Convergence

Ar-Ar

Table S1: Convergence with basis set size for Ar-Ar system using BLYP functional. Energies in $kcal/mol$

Property	def2-SVP	def2-TZVP	def2-TZVP(-f)	def2-QZVP
ΔE_{int}	-0.25	-0.14	-0.14	-0.15
ΔE_{orb}	-0.01	-0.04	-0.04	-0.05
ΔE_{elstat}	-0.11	-0.17	-0.17	-0.18
ΔE_{Pauli}	0.39	0.58	0.58	0.59
ΔE_{disp}	-0.52	-0.52	-0.52	-0.52
ΔE_1^{orb}	-0.01	-0.02	-0.02	-0.02
ΔE_2^{orb}	0.00	-0.02	-0.02	-0.02

Table S2: Convergence with basis set size for Ar-Ar system using B3LYP functional. Energies in $kcal/mol$

Property	def2-SVP	def2-TZVP	def2-TZVP(-f)	def2-QZVP
ΔE_{int}	-0.24	-0.20	-0.20	-0.21
ΔE_{orb}	-0.01	-0.03	-0.03	-0.04
ΔE_{elstat}	-0.18	-0.28	-0.28	-0.29
ΔE_{Pauli}	0.37	0.54	0.54	0.55
ΔE_{disp}	-0.43	-0.43	-0.43	-0.43
ΔE_1^{orb}	0.00	-0.01	-0.01	-0.02
ΔE_2^{orb}	0.00	-0.01	-0.01	-0.02

Table S3: Convergence with basis set size for Ar-Ar system using B2PYLP functional. Energies in $kcal/mol$

Property	def2-SVP	def2-TZVP	def2-TZVP(-f)	def2-QZVP
ΔE_{int}	-0.11	-0.13	-0.12	-0.18
ΔE_{orb}	0.00	-0.02	-0.02	-0.03
ΔE_{elstat}	-0.27	-0.46	-0.46	-0.47
ΔE_{Pauli}	0.43	0.66	0.66	0.68
ΔE_{disp}	-0.22	-0.22	-0.22	-0.22
ΔE_C^{MP2}	-0.04	-0.09	-0.09	-0.14
ΔE_1^{orb}	0.00	-0.01	-0.01	-0.01
ΔE_2^{orb}	0.00	-0.01	-0.01	-0.01

Ar-Li^+

Table S4: Convergence with basis set size for Ar-Li^+ system using BLYP functional. Energies in $kcal/mol$

Property	def2-SVP	def2-TZVP	def2-TZVP(-f)	def2-QZVP
ΔE_{int}	-9.16	-7.40	-7.27	-8.33
ΔE_{orb}	-7.98	-9.85	-9.72	-11.66
ΔE_{elstat}	-4.69	-0.86	-0.86	0.06
ΔE_{Pauli}	4.42	4.22	4.22	4.18
ΔE_{disp}	-0.92	-0.92	-0.92	-0.92
ΔE_1^{orb}	-5.68	-6.84	-6.77	-7.55
ΔE_2^{orb}	-1.08	-1.44	-1.41	-1.98
ΔE_3^{orb}	-1.08	-1.44	-1.41	-1.98

Table S5: Convergence with basis set size for Ar-Li^+ system using B3LYP functional. Energies in $kcal/mol$

Property	def2-SVP	def2-TZVP	def2-TZVP(-f)	def2-QZVP
ΔE_{int}	-8.48	-6.98	-6.86	-8.13
ΔE_{orb}	-7.66	-9.54	-9.41	-11.33
ΔE_{elstat}	-4.65	-1.12	-1.12	-0.44
ΔE_{Pauli}	4.59	4.45	4.45	4.41
ΔE_{disp}	-0.77	-0.77	-0.77	-0.77
ΔE_1^{orb}	-5.48	-6.63	-6.56	-7.36
ΔE_2^{orb}	-1.02	-1.39	-1.36	-1.91
ΔE_3^{orb}	-1.02	-1.39	-1.36	-1.91

Table S6: Convergence with basis set size for Ar-Li^+ system using B2PYLP functional. Energies in $kcal/mol$

Property	def2-SVP	def2-TZVP	def2-TZVP(-f)	def2-QZVP
ΔE_{int}	-7.58	-6.24	6.09	-7.60
ΔE_{orb}	-7.22	-9.11	-8.98	-10.87
ΔE_{elstat}	-4.89	-1.65	-1.65	-1.17
ΔE_{Pauli}	5.13	5.05	5.05	5.02
ΔE_{disp}	-0.41	-0.41	-0.41	-0.41
ΔE_C^{MP2}	-0.19	-0.12	-0.09	-0.17
ΔE_1^{orb}	-5.17	-6.32	-6.25	-7.06
ΔE_2^{orb}	-0.96	-1.33	-1.30	-1.83
ΔE_3^{orb}	-0.96	-1.33	-1.30	-1.83

Be-Be

Table S7: Convergence with basis set size for Be-Be system using BLYP functional.Energies in $kcal/mol$

Property	def2-SVP	def2-TZVP	def2-TZVP(-f)	def2-QZVP
ΔE_{int}	-8.88	-8.37	-8.37	-8.72
ΔE_{orb}	-32.47	-31.70	-31.70	-32.04
ΔE_{elstat}	-18.59	-17.96	-17.96	-17.91
ΔE_{Pauli}	44.79	43.90	43.90	43.84
ΔE_{disp}	-2.61	-2.61	-2.61	-2.61
ΔE_1^{orb}	-21.02	-20.13	-20.13	-20.35
ΔE_2^{orb}	-11.46	-11.57	-11.57	-11.70

Table S8: Convergence with basis set size for Be-Be system using B3LYP functional.Energies in $kcal/mol$

Property	def2-SVP	def2-TZVP	def2-TZVP(-f)	def2-QZVP
ΔE_{int}	-6.35	-5.93	-5.93	-6.23
ΔE_{orb}	-29.89	-29.10	-29.10	-29.40
ΔE_{elstat}	-31.50	-30.41	-30.41	-30.35
ΔE_{Pauli}	57.22	55.75	55.75	55.69
ΔE_{disp}	-2.17	-2.17	-2.17	-2.17
ΔE_1^{orb}	-19.56	-18.68	-18.68	-18.86
ΔE_2^{orb}	-10.33	-10.42	-10.42	-10.54

Table S9: Convergence with basis set size for Be-Be system using B2PYLP functional. Energies in $kcal/mol$

Property	def2-SVP	def2-TZVP	def2-TZVP(-f)	def2-QZVP
ΔE_{int}	-2.03	-2.74	-2.74	-3.40
ΔE_{orb}	-26.08	-25.38	-25.38	-25.65
ΔE_{elstat}	-53.51	-51.60	-51.60	-51.53
ΔE_{Pauli}	80.63	78.19	78.19	78.12
ΔE_{disp}	-1.11	-1.11	-1.11	-1.11
ΔE_C^{MP2}	-1.95	-2.84	-2.84	-3.24
ΔE_1^{orb}	-17.28	-16.49	-16.49	-16.63
ΔE_2^{orb}	-8.80	-8.90	-8.90	-9.02

HF-HF

Table S10: Convergence with basis set size for HF-HF system using BLYP functional. Energies in $kcal/mol$

Property	def2-SVP	def2-TZVP	def2-TZVP(-f)	def2-QZVP
ΔE_{int}	-5.10	-4.75	-4.78	-4.53
ΔE_{orb}	-3.11	-3.30	-3.30	-3.47
ΔE_{elstat}	-8.44	-7.33	-7.34	-6.76
ΔE_{Pauli}	6.91	6.33	6.32	6.16
ΔE_{disp}	-0.45	-0.45	-0.45	-0.45
ΔE_1^{orb}	-2.78	-2.87	-2.87	-2.94
ΔE_2^{orb}	-0.14	-0.16	-0.16	-0.17
ΔE_3^{orb}	-0.08	-0.10	-0.10	-0.15

Table S11: Convergence with basis set size for HF-HF system using B3LYP functional. Energies in $kcal/mol$

Property	def2-SVP	def2-TZVP	def2-TZVP(-f)	def2-QZVP
ΔE_{int}	-5.40	-5.04	-5.07	-4.87
ΔE_{orb}	-2.75	-2.95	-2.95	-3.13
ΔE_{elstat}	-9.93	-8.78	-8.79	-8.26
ΔE_{Pauli}	7.66	7.05	7.03	6.89
ΔE_{disp}	-0.37	-0.37	-0.37	-0.37
ΔE_1^{orb}	-2.43	-2.53	-2.53	-2.6
ΔE_2^{orb}	-0.13	-0.15	-0.15	-0.17
ΔE_3^{orb}	-0.08	-0.11	-0.11	-0.15

Table S12: Convergence with basis set size for HF-HF system using B2PYLP functional. Energies in $kcal/mol$

Property	def2-SVP	def2-TZVP	def2-TZVP(-f)	def2-QZVP
ΔE_{int}	-5.11	-4.83	-4.84	-4.75
ΔE_{orb}	-2.29	-2.49	-2.49	-2.66
ΔE_{elstat}	-11.97	-10.82	-10.82	-10.35
ΔE_{Pauli}	9.30	8.72	8.70	8.56
ΔE_{disp}	-0.16	-0.16	-0.16	-0.16
ΔE_C^{MP2}	0.01	-0.08	-0.06	0.15
ΔE_1^{orb}	-1.97	-2.06	-2.07	-2.15
ΔE_2^{orb}	-0.13	-0.15	-0.15	-0.17
ΔE_3^{orb}	-0.10	-0.13	-0.12	-0.14

H_2O-H_2O

Table S13: Convergence with basis set size for H_2O-H_2O system using BLYP functional. Energies in *kcal/mol*

Property	def2-SVP	def2-TZVP	def2-TZVP(-f)	def2-QZVP
ΔE_{int}	-5.11	-5.25	-5.29	-4.85
ΔE_{orb}	-3.33	-3.45	-3.45	-3.55
ΔE_{elstat}	-9.78	-9.12	-9.14	-8.40
ΔE_{Pauli}	8.77	8.08	8.07	7.87
ΔE_{disp}	-0.77	-0.77	-0.77	-0.77
ΔE_1^{orb}	-2.93	-2.96	-2.97	-3.00
ΔE_2^{orb}	-0.17	-0.20	-0.20	-0.22
ΔE_3^{orb}	-0.05	-0.10	-0.09	-0.12

Table S14: Convergence with basis set size for H_2O-H_2O system using B3LYP functional. Energies in *kcal/mol*

Property	def2-SVP	def2-TZVP	def2-TZVP(-f)	def2-QZVP
ΔE_{int}	-5.50	-5.56	-5.60	-5.17
ΔE_{orb}	-2.98	-3.11	-3.11	-3.21
ΔE_{elstat}	-12.10	-11.23	-11.25	-10.52
ΔE_{Pauli}	10.21	9.40	9.39	9.19
ΔE_{disp}	-0.63	-0.63	-0.63	-0.63
ΔE_1^{orb}	-2.60	-2.64	-2.64	-2.68
ΔE_2^{orb}	-0.16	-0.20	-0.20	-0.22
ΔE_3^{orb}	-0.05	-0.09	-0.09	-0.12

Table S15: Convergence with basis set size for H_2O-H_2O system using B2PLYP functional. Energies in *kcal/mol*

Property	def2-SVP	def2-TZVP	def2-TZVP(-f)	def2-QZVP
ΔE_{int}	-5.15	-5.33	-5.34	-5.05
ΔE_{orb}	-2.55	-2.67	-2.68	-2.77
ΔE_{elstat}	-15.40	-14.30	-14.32	-13.61
ΔE_{Pauli}	13.15	12.20	12.18	11.99
ΔE_{disp}	-0.28	-0.28	-0.28	-0.28
ΔE_C^{MP2}	-0.07	-0.27	-0.24	-0.37
ΔE_1^{orb}	-2.15	-2.19	-2.19	-2.24
ΔE_2^{orb}	-0.19	-0.23	-0.23	-0.24
ΔE_3^{orb}	-0.06	-0.08	-0.08	-0.11

Dirhodium(II)-Carbene

Table S16: Convergence with basis set size for Dirhodium(II)-Carbene system using BLYP functional. Energies in $kcal/mol$

Property	def2-SVP	def2-TZVP	def2-TZVP(-f)	def2-QZVP
ΔE_{int}	-66.36	-66.48	-66.13	-67.71
ΔE_{orb}	-118.99	-120.23	-119.83	-121.48
ΔE_{elstat}	-118.09	-116.32	-116.30	-116.07
ΔE_{Pauli}	192.81	192.16	192.09	191.94
ΔE_{disp}	-22.09	-22.09	-22.09	-22.09
ΔE_C^{MP2}	0.00	0.00	0.00	0.00
ΔE_1^{orb}	-36.99	-35.12	-35.00	-34.93
ΔE_2^{orb}	-63.52	-65.49	-65.30	-66.56
ΔE_3^{orb}	-6.82	-7.03	-7.00	-7.07

Table S17: Convergence with basis set size for Dirhodium(II)-Carbene system using B3LYP functional. Energies in $kcal/mol$

Property	def2-SVP	def2-TZVP	def2-TZVP(-f)	def2-QZVP
ΔE_{int}	-60.08	-60.11	-59.79	-61.16
ΔE_{orb}	-107.79	-109.05	-108.68	-110.13
ΔE_{elstat}	-157.75	-155.89	-155.88	-155.63
ΔE_{Pauli}	224.18	223.56	223.49	223.31
ΔE_{disp}	-18.72	-18.72	-18.72	-18.72
ΔE_C^{MP2}	0.00	0.00	0.00	0.00
ΔE_1^{orb}	-66.96	-66.38	-66.21	-66.59
ΔE_2^{orb}	-23.87	-24.60	-24.47	-25.14
ΔE_3^{orb}	-6.62	-6.88	-6.86	-6.92

Table S18: Convergence with basis set size for Dirhodium(II)-Carbene system using B2PLYP functional. Energies in $kcal/mol$

**Property**	**def2-SVP**	**def2-TZVP**	**def2-TZVP(-f)**	**def2-QZVP**
ΔE_{int}	-56.21	-59.58	-59.58	-62.45
ΔE_{orb}	-95.76	-97.08	-97.08	-97.81
ΔE_{elstat}	-221.53	-219.79	-219.79	-219.47
ΔE_{Pauli}	285.73	285.44	285.44	285.12
ΔE_{disp}	-9.80	-9.80	-9.80	-9.80
ΔE_C^{MP2}	-14.85	-18.35	-18.35	-20.50
ΔE_1^{orb}	-64.12	-63.73	-63.73	-63.84
ΔE_2^{orb}	-15.52	-16.13	-16.13	-16.47
ΔE_3^{orb}	-7.14	-7.53	-7.53	-7.60

Natural Orbitals for Chemical Valence

Here are presented the deformation densities for the studied systems along with their corresponding NOCV eigenvalue (v_i) and orbital interaction energy contribution ($\Delta E_{orb,i}$).

Dirhodium(II)-Carbene

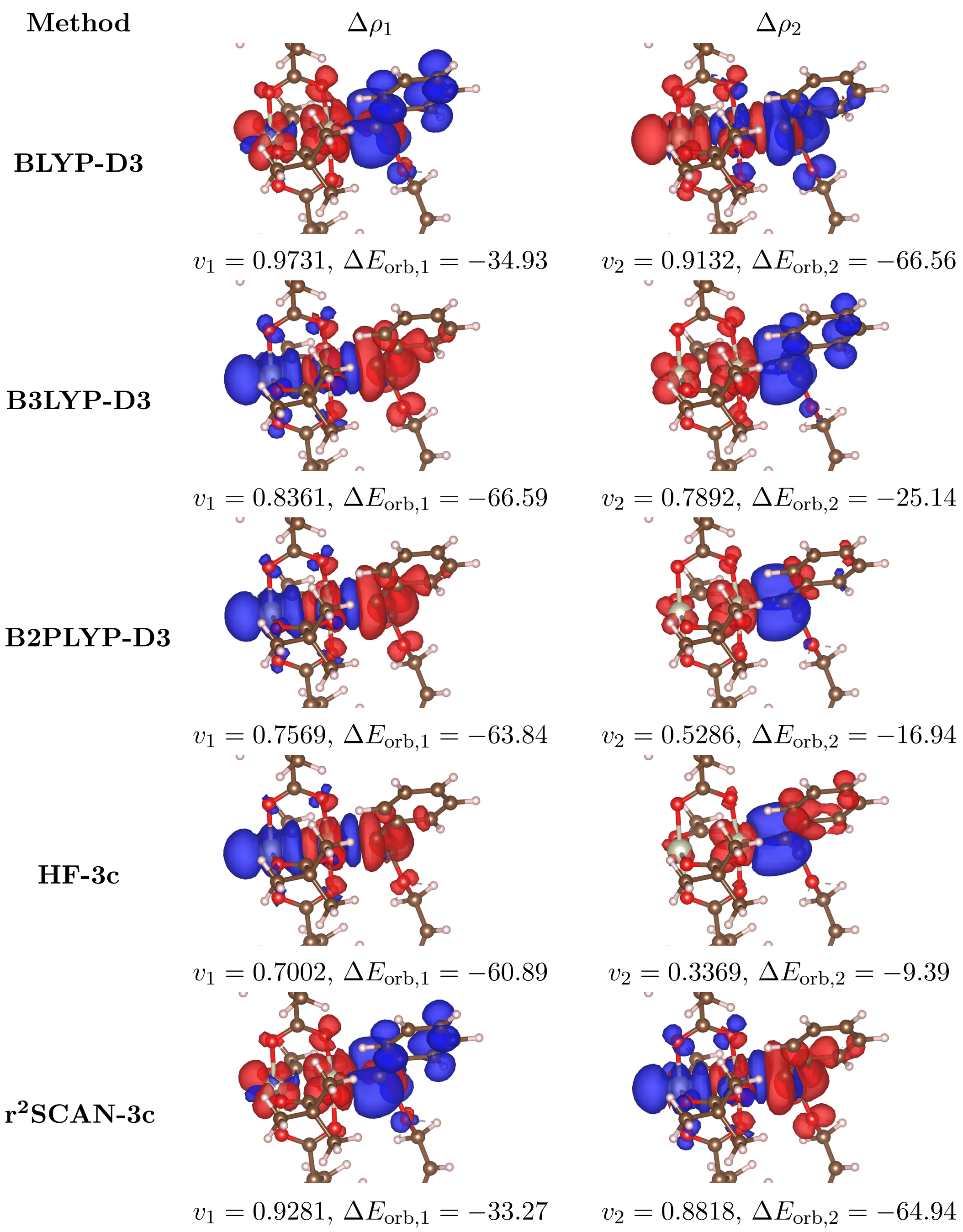

Figure S2: First two NOCV deformation densities $\Delta\rho_1$ and $\Delta\rho_2$ for the Dirhodium(II)–Carbene system across five electronic structure methods. Each row corresponds to a functional, with the corresponding NOCV eigenvalues (v_i) and orbital interaction energy contributions ($\Delta E_{\mathrm{orb},i}$ in kcal/mol) shown below each deformation density. All deformation densities are visualized at an isosurface level of 5×10^{-4} e/Bohr^3.

MP2 Correlation Electron Density Contribution $\Delta\Delta\rho_C^{MP2}$

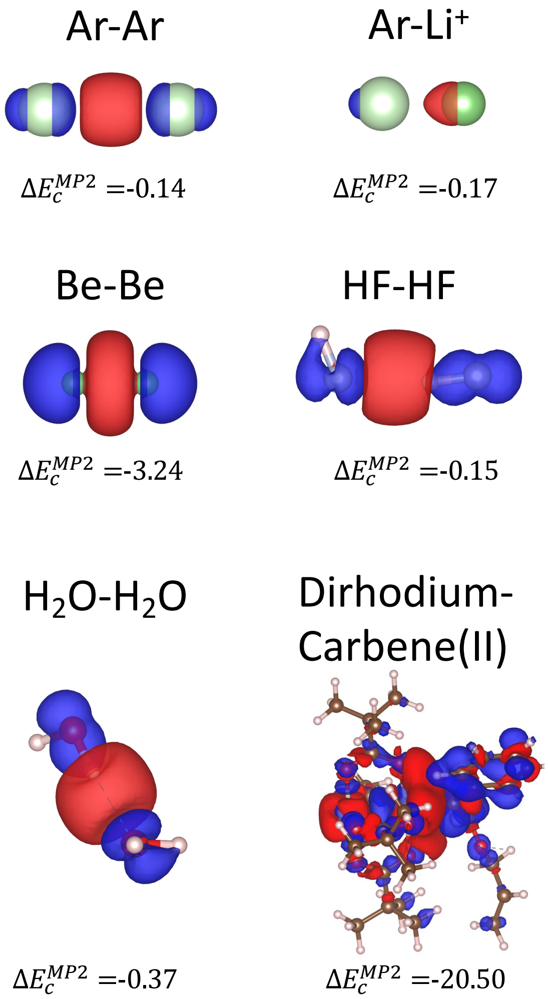

Figure S3: Electron density contributions from MP2 correlation, computed using the relaxed MP2 electron density and shown with the same isosurface levels as for the NOCV deformation densities: 2×10^{-5} e/Bohr^3 (Ar dimer), 2×10^{-4} e/Bohr^3 (Ar–Li$^+$, HF dimer), 5×10^{-4} e/Bohr^3 (Be dimer, dirhodium(II)–carbene complex), and 1×10^{-4} e/Bohr^3 (water dimer). The MP2 correlation energy contributions to the interaction energy are also reported.

Structures

HF-HF

```
H -0.38796278517536  0.86330637901020 -0.00000977491860
F -0.04061354010285  0.00605168994919  0.00003560268516
H  1.78453684015533 -0.06619212709085 -0.00007106862474
F  2.70496248512288  0.05222805813148  0.00004524085818
```

H_2O-H_2O

```
O  0.00734121594622  0.01158551260826  0.01667101625365
H  0.96920243723402  0.01150817186684 -0.00782003942165
H -0.23653892850061  0.94167735415384 -0.01792651828719
O -0.98963357612938 -1.29467086163939  2.41546057115780
H -0.69832831461969 -0.90500029812740  1.57798060549298
H -1.52574840471901 -2.04779465760851  2.15509691587116
```

Dirhodium-Carbene

```
C  -2.48912703135947 -1.28864370172808  0.51478710370416
O  -1.81949811270045 -2.35034443615123  0.38073018617804
Rh  0.14386546702445 -2.24446295144206 -0.23390379942505
Rh -0.14586674688816  0.19335758532148 -0.40558583960381
N  -0.67271173888146 -0.16579477495539 -2.31956365653649
C  -0.69806316440088 -1.36581028843004 -2.84923476349386
O  -0.42625304164230 -2.43079743357692 -2.19173336468984
C  -1.05962259522395 -1.54748885271756 -4.30228556183561
H  -1.29293553839986 -0.59488142587369 -4.79626297618764
H  -1.92770248516812 -2.22082803792983 -4.37558267881765
```

H	-0.21904417359422	-2.03326232453293	-4.82195262152780
H	-0.87272423085141	0.62161460847307	-2.93345663968443
O	-2.07912957535443	-0.12303153001916	0.20817463322398
O	1.82025746895981	0.28369472161160	-0.98773732023166
C	2.51222708394725	-0.78150324134430	-1.08116762367109
O	2.09150032061695	-1.94548870441410	-0.83372306759110
O	0.45811440577757	0.26605276409467	1.59850943273312
C	0.73504049370318	-0.80070188029769	2.22940207131918
O	0.70161561546767	-1.96231900327987	1.73195900313270
C	3.95951415544217	-0.59875754848304	-1.55398662935654
C	4.74311771421485	-1.90996408380459	-1.42189979482788
C	4.61789502270946	0.50471933445631	-0.70440979800476
C	3.90083294808980	-0.16138670925635	-3.03323626475795
H	5.77648042817388	-1.75765470736572	-1.77138078497792
H	4.28807486682547	-2.71115960081667	-2.02219820882658
H	4.77900799508884	-2.24823796487266	-0.37502048771072
H	4.08788928033174	1.45972616417895	-0.81815040167109
H	5.66214500456624	0.64677797938664	-1.02486827489358
H	4.62032401654212	0.23154410591076	0.36341007975151
H	4.92117553167111	0.00278878535458	-3.41436280600054
H	3.33385575537000	0.77376469453940	-3.14329349384581
H	3.42092044652249	-0.93454317864873	-3.65447447100628
C	1.13585364419105	-0.63589594144459	3.70086505558377
C	-0.02760042085704	0.06344808789689	4.43254936579166
C	2.39631135603389	0.25132932342553	3.74892876337964
C	1.42216344288045	-1.99676497003784	4.34601062158783
H	0.22641206555760	0.19481008419648	5.49632456902466

H	-0.94995656173169	-0.53603512107338	4.37055549245406
H	-0.23029098947301	1.05280153953354	3.99787109150703
H	0.53656796269795	-2.64894497311605	4.31255070276396
H	1.70547553872003	-1.85198818230991	5.40057616116068
H	2.24602801700502	-2.51672020299491	3.83484796764462
H	2.19704164055049	1.24117916102635	3.31348278101067
H	3.22691949777496	-0.21182941319952	3.19169948051688
H	2.71749205075440	0.38675794812119	4.79393669070985
C	-3.87663685899666	-1.37723292065210	1.16148415593260
C	-4.48118934395605	-2.77100041857862	0.93968251515042
C	-4.80533878265562	-0.29580611743282	0.58770775169597
C	-3.65481664848190	-1.13035880467422	2.67177904680017
H	-4.61065946349922	-2.98047722284731	-0.13388703358833
H	-5.46963422545687	-2.82486109695613	1.42228001696174
H	-3.84193596467925	-3.55626247334431	1.36675456832485
H	-4.61652652120963	-1.20565695135704	3.20354258594021
H	-3.23783862369293	-0.12776305009560	2.85247334702897
H	-2.96404298067997	-1.87525251554834	3.09700710924461
H	-5.79310477036935	-0.36422092516223	1.07003518240994
H	-4.94384735061021	-0.42677051612658	-0.49734790376817
H	-4.40114406347431	0.71078260444241	0.76358631738653
C	-0.37986094162333	2.16162788046218	-0.52504006437922
C	-1.07394872212933	3.02053261764084	0.38000511967398
C	-1.20213992218891	4.41665221536765	0.11374577420249
C	-1.86945567296024	5.24929229332251	1.00021260665303
C	-2.41957814535668	4.71731507368953	2.17750362821437
C	-2.30247414222386	3.35048481241353	2.46552094752400

C	-1.64268816139210	2.50669081748346	1.58092829181803
H	-1.96439741386635	6.31569877139038	0.78457965035718
H	-2.94181067810323	5.37655912211868	2.87578651269330
H	-2.72965493024520	2.94615316361919	3.38584481479727
H	-1.54434815364341i8	1.44760298029611	1.79805191016035
H	-0.77397680320346	4.83662486882236	-0.79873029009978
C	0.23462534110328	2.75434525259643	-1.73497728526424
O	-0.28904363013349	2.68855089528429	-2.83340442813741
O	1.42687330352232	3.29223473289317	-1.49635229241391
C	2.18171475580535	3.75629472615259	-2.64837929780731
C	3.49423292422493	4.27345885716388	-2.15853550467335
H	2.30217579963096	2.92227501681614	-3.35515855493908
H	1.59201690415825	4.54602791686697	-3.14208216739889
C	4.67510028683220	3.82552234706679	-2.59339839425118
H	3.44330777411335	5.07858708655829	-1.41555731889449
H	4.74231153055304	3.01548240820845	-3.32710400584773
H	5.61498887970346	4.25272133098874	-2.23213801220927
